# Diversity in Protein Glycosylation among Insect Species

**DOI:** 10.1371/journal.pone.0016682

**Published:** 2011-02-23

**Authors:** Gianni Vandenborre, Guy Smagghe, Bart Ghesquière, Gerben Menschaert, Rameshwaram Nagender Rao, Kris Gevaert, Els J. M. Van Damme

**Affiliations:** 1 Laboratory of Agrozoology, Department of Crop Protection, Faculty of Bioscience Engineering, Ghent University, Ghent, Belgium; 2 Laboratory of Biochemistry and Glycobiology, Department of Molecular Biotechnology, Faculty of Bioscience Engineering, Ghent University, Ghent, Belgium; 3 Department of Medical Protein Research, VIB, Ghent, Belgium; 4 Department of Biochemistry, Faculty of Medicine and Health Sciences, Ghent University, Ghent, Belgium; 5 Laboratory for Bioinformatics and Computational Genomics, Department of Molecular Biotechnology, Faculty of Bioscience Engineering, Ghent University, Ghent, Belgium; AgroParisTech, France

## Abstract

**Background:**

A very common protein modification in multicellular organisms is protein glycosylation or the addition of carbohydrate structures to the peptide backbone. Although the Class of the Insecta is the largest animal taxon on Earth, almost all information concerning glycosylation in insects is derived from studies with only one species, namely the fruit fly *Drosophila melanogaster*.

**Methodology/Principal Findings:**

In this report, the differences in glycoproteomes between insects belonging to several economically important insect orders were studied. Using GNA (*Galanthus nivalis* agglutinin) affinity chromatography, different sets of glycoproteins with mannosyl-containing glycan structures were purified from the flour beetle (*Tribolium castaneum*), the silkworm (*Bombyx mori*), the honeybee (*Apis mellifera*), the fruit fly (*D. melanogaster*) and the pea aphid (*Acyrthosiphon pisum*). To identify and characterize the purified glycoproteins, LC-MS/MS analysis was performed. For all insect species, it was demonstrated that glycoproteins were related to a broad range of biological processes and molecular functions. Moreover, the majority of glycoproteins retained on the GNA column were unique to one particular insect species and only a few glycoproteins were present in the five different glycoprotein sets. Furthermore, these data support the hypothesis that insect glycoproteins can be decorated with mannosylated *O*-glycans.

**Conclusions/Significance:**

The results presented here demonstrate that oligomannose *N*-glycosylation events are highly specific depending on the insect species. In addition, we also demonstrated that protein *O*-mannosylation in insect species may occur more frequently than currently believed.

## Introduction

Glycosylation is the covalent attachment of an oligosaccharide chain to a protein backbone and is considered to be a very common protein modification. The structure and size of the carbohydrate chain can be very diverse and can alter the physico-chemical characteristics of a protein. Two major types of glycosylation, referred to as *N*- and *O*-linked glycosylation, can be distinguished. *N*-glycans are attached to Asn residues of the peptide backbone while *O*-glycans are connected to Ser or Thr residues. Only in recent years, it has been acknowledged that glycosylation of proteins modulates various processes such as subcellular localization, protein quality control, cell-cell recognition and cell-matrix binding events. In turn, these important functions control developmental processes such as embryogenesis or organogenesis [1]–[6]. Although the overall importance of glycosylation is recognized nowadays, the different types of glycosylated proteins in an organism are mostly unknown indicating that the full range of biological and cellular functions is still not fully understood. Deciphering the complexities in biosynthesis and function of glycoproteins in multicellular organisms is a major challenge for the coming decade.

Insects are without any doubt the largest animal taxon found on Earth accounting for more than half of all known living species [7]. Their unprecedented evolutionary success is the result of an enormous genetic and phenotypic diversification allowing insect species to adapt to a wide variety of ecological niches and environmental challenges. For example, the genetic diversity within one insect order (*e.g.* Diptera) is already much wider than between distant vertebrates such as human and zebrafish, spanning a whole phylum [8], [9]. Because insects are the most diverse organisms in the history of life, they should provide profound insights into diversification of glycobiology in general and differences of glycosylation in particular. To date, almost all information concerning glycobiology in insects was obtained from studies with the fruit fly, *Drosophila melanogaster* (Diptera), the best studied insect laboratory model organism. For *D. melanogaster*, different glycosyltransferases and glycosylhydrolases which are responsible for synthesis and trimming of *N*-glycans have been reported suggesting the presence of multiple glycan structures on glycoproteins [10]–[13]. Moreover, at least 42 discrete *N*-glycans have been identified recently in *D. melanogaster*, mostly containing oligomannose and core fucosylated paucimannosidic *N*-glycans [14]–[17]. Considering the broad diversity among insect species, it can be expected that the diversification in glycan patterns will even be more extensive when analyzing glycosylation patterns in different insect species.

In this study, the functional diversity of glycoproteins was studied for insect species belonging to five important insect orders. We selected four insects with a complete metamorphosis, the flour beetle *Tribolium castaneum* (Coleoptera), the silkworm *Bombyx mori* (Lepidoptera), the honeybee *Apis mellifera* (Hymenoptera) and the fruit fly *D. melanogaster* (Diptera), as well as one insect species with an incomplete metamorphosis, the pea aphid *Acyrthosiphon pisum* (Hemiptera). In addition to this wide selection in insect diversity, several insect species are good representatives for economically important pest insects such as caterpillars, beetles or aphids, while the honeybee belongs to the group of beneficial insects that are essential for pollination. Flies and mosquitoes, on the other hand, are important transmitters of many (human) diseases. Because protein modifications such as glycosylation are not directly encoded by the genomic code, glycosylation in insects was studied at the proteomics level. Recent developments in high-throughput technology for studying proteomes and the public availability of the genome data of different insect species allowed a comparative study of the glycoproteins present in the different insect species. Lectin affinity chromatography using the snowdrop lectin (*Galanthus nivalis* agglutinin, GNA) was used to selectively purify different sets of mannosylated glycoproteins from different insect species. Subsequently, the purified glycoproteins were identified with LC-MS/MS and characterized according to biological or molecular function. To our knowledge, this is the first report that presents a comparative study of the glycoproteomes present in different insect species. Studying glycoproteomes in different insect species should ultimately result in the development of a more holistic understanding of the importance of glycobiology in insects.

## Results

### Purification and identification of glycoproteins from insects

To study the functional differences in glycoprotein sets derived from insect species belonging to different insect orders, glycoproteins were captured using lectin affinity chromatography based on the snowdrop lectin GNA (Figure S1). As shown by the glycan microarray experiments conducted by the Consortium for Functional Glycomics, GNA has a high selectivity for oligomannose *N*-glycans [18] that were previously shown to be the most abundant class of *N*-glycans present in insects. The percentage of proteins retained on the GNA column was less than 5% of the total amount of proteins (based on protein concentration estimations using Bradford) for all five insect species. Peptide identification using LC-MS/MS, resulted in 161, 64, 116, 142 and 245 unique (glyco)proteins for *T. castaneum*, *B. mori*, *A. mellifera*, *D. melanogaster* and *A. pisum*, respectively (Table 1). Putative *N*-glycosylation sites were present on 81%, 77%, 75%, 83% and 89% of the glycoproteins from *T. castaneum*, *B. mori*, *A. mellifera*, *D. melanogaster* and *A. pisum*, respectively (Table 1). This suggests that for all insect species at least 11% of the glycoproteins were purified in an *N*-glycan independent way.

**Table 1:** Number of glycoproteins purified by GNA affinity chromatography for the different insect species.

Insect species	Insect order	No of proteins in database	No of generated spectra	No of peptides identified	No of identified proteins	No of putative N-glycosylated proteins
*Tribolium castaneum*	Coleoptera	16.645	3744	572	161	130
*Bombyx mori*	Lepidoptera	14.623	3749	118	64	49
*Apis mellifera*	Hymenoptera	10.157	3496	381	116	87
*Drosophila melanogaster*	Diptera	21.317	3744	655	142	118
*Acyrthosiphon pisum*	Hemiptera	34.821	3745	788	245	218

After identification of the different sets of glycoproteins, InterProScan was used to detect functional domains, protein regions or protein signatures in the individual polypeptides for further annotation (Tables S1, S2, S3, S4, S5). Subsequently, a protein abundance index (emPAI) was calculated to detect the polypeptide sequences that were highly abundant among the captured glycoproteins (Tables S1, S2, S3, S4, S5). Among the identified glycoproteins typical membrane proteins such as laminin, cadherin, contactin, chaoptin or C-type lectins were found to be abundantly present (Table S1, S2, S3, S4, S5). Also many leucine-rich repeat transmembrane proteins which are known to contain several glycans on their extracellular part were detected. Transport proteins were lipoproteins, hemocyanin or ferritin. Also vitellogenin, which is a known glycolipoprotein present in the fat body of adult insects and important for reproduction, was detected in *T. castaneum*, *D. melanogaster* and *A. pisum*. Next to the typical receptor proteins or secreted proteins, many GNA-captured glycoproteins were identified as metabolic enzymes (e.g. dehydrogenases, proteases and amylases), ribosomal proteins or intracellular structural proteins (e.g. actin, tubulin). Because many of these proteins are synthesized on free ribosomes and, consequently, do not enter the ER-Golgi pathway, oligomannosidic *N*-glycans are thought to be absent from these proteins. Therefore the putative *N*-glycosylation sites found on the peptide backbone of these proteins (Tables S1, S2, S3, S4, S5) may not be functional.

Comparing the insect specific glycoprotein sets, major differences in both glycoprotein diversity and quantity were observed (Table S6). When comparing a particular protein annotation such as leucine-rich transmembrane protein between the different insect species, 15, 4 and 1 glycoprotein(s) were detected for *A. pisum*, *T. castaneum* and *D. melanogaster*, respectively, while for *A. mellifera* and *B. mori* no leucine-rich membrane proteins were found (Table 2). From the 260 different protein annotations found over the different sets of insect-specific glycoproteins, 62% (161 protein annotations) were associated with only one particular insect species while 1.5% of the proteins (only 4 protein annotations) were detected for all five insect species (Tables 2 and S6). This remarkable diversity in glycoproteome profiles between insect species may reveal underlying differences that can influence certain biological processes.

**Table 2:** Summary table for the number of distinct (glyco)proteins found in at least three different insect species.

	Protein description	*A. pisum*	*D. melanogaster*	*A. mellifera*	*B. mori*	*T. castaneum*
1	2-OXOGLUTARATE DEHYDROGENASE	0	1	1	0	1
2	3-HYDROXYACYL-COA DEHYROGENASE	1	1	1	0	1
3	ACETYL-COA C-ACYLTRANSFERASE	1	0	1	0	1
4	ACTIN	2	1	2	2	1
5	ALDEHYDE DEHYDROGENASE	2	1	0	0	2
6	ALPHA-AMYLASE	3	0	1	1	2
7	ALPHA-GALACTOSIDASE	5	1	0	0	1
8	ALPHA-MANNOSIDASE	4	2	0	0	1
9	AMINOPEPTIDASE	4	5	1	1	1
10	DIPEPTIDYL CARBOXYPEPTIDASE	0	2	0	1	1
11	ARGININE KINASE	0	1	1	0	1
12	ASPARTATE AMMONIA LYASE	0	0	1	1	1
13	ATP SYNTHASE SUBUNIT	4	2	6	2	5
14	BETA-HEXOSAMINIDASE	3	1	2	0	1
15	CADHERIN	1	0	1	0	1
16	CARBOXYLESTERASE	4	1	0	1	1
17	CATHEPSIN	1	0	1	0	1
18	CONTACTIN	1	1	1	0	1
19	ELONGATION FACTOR 1-ALPHA	1	0	1	0	1
20	GLYCERALDEHYDE 3-PHOSPHATE DEHYDROGENASE	1	0	1	0	1
21	GLYCOGEN DEBRANCHING ENZYME	2	1	1	0	0
22	GLYCOGEN PHOSPHORYLASE	1	0	1	1	1
23	HEAT SHOCK PROTEINS	5	0	4	1	7
24	HEMOCYANIN	0	1	2	2	6
25	ISOCITRATE DEHYDROGENASE	1	2	1	0	0
26	LAMININ	5	4	0	1	4
27	LEUCINE-RICH TRANSMEMBRANE PROTEIN	15	1	0	0	4
28	LOW DENSITY LIPOPROTEIN RECEPTOR	1	1	1	0	2
29	MUCIN	0	1	2	2	0
30	MYOSIN	1	1	0	2	0
31	PEROXIREDOXIN	0	0	1	1	1
32	PHOSPHOFRUCTOKINASE	1	1	1	0	0
33	PROTEASE S28 PRO-X CARBOXYPEPTIDASE	2	1	0	0	1
34	PROTEIN DISULFIDE ISOMERASE	3	1	0	1	1
35	RIBOSOMAL PROTEINS	18	14	10	5	22
36	SERINE CARBOXYPEPTIDASE	2	1	0	0	1
37	SERINE PROTEASE INHIBITOR, SERPIN	4	1	0	1	4
38	SERINE PROTEASE-RELATED	2	7	0	7	0
39	TRANSKETOLASE	1	0	1	0	1
40	TREHALOSE-6-PHOSPHATE SYNTHASE	1	1	1	0	0
41	TROPOMYOSIN	1	0	0	1	1
42	TUBULIN	4	4	4	1	0
44	VITELLOGENIN-RELATED	1	2	0	0	4

### Functional classification of glycoproteins from insects

**Figure 1:**
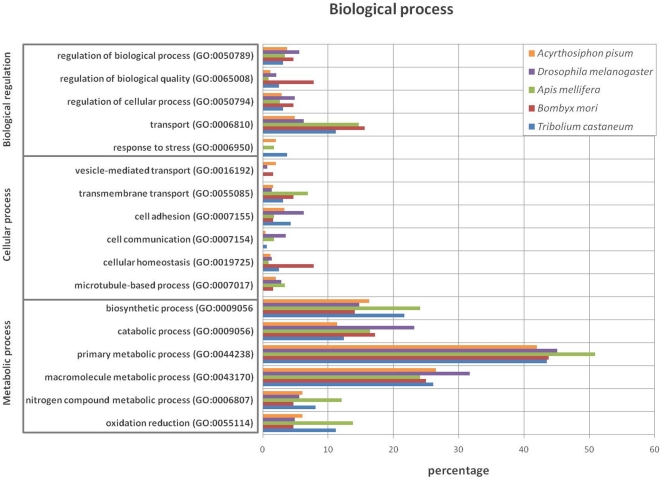
Classification according to biological process of the GNA binding glycoproteins from *A. pisum, D. melanogaster*, *A. mellifera, B. mori* and *T. castaneum* using the WEGO resources.

**Figure 2:**
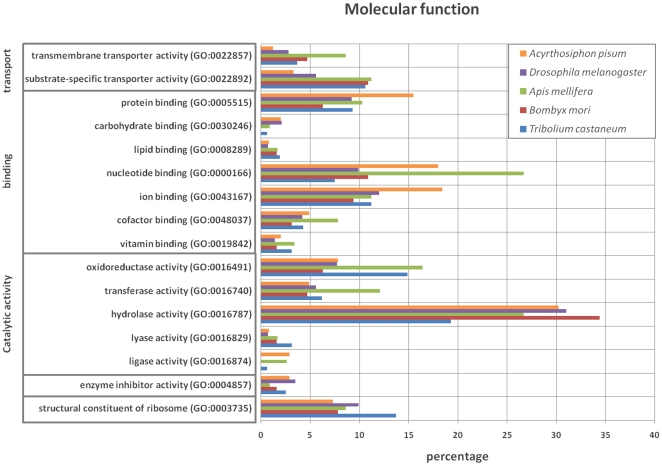
Classification according to molecular function of the GNA binding glycoproteins from *A. pisum, D. melanogaster*, *A. mellifera, B. mori* and *T. castaneum* using the WEGO resources.

After identification and annotation of the different polypeptides, the different sets of glycoproteins were classified according to biological process and molecular function using the web-based WEGO plotting tool (Figures 1 and 2). Hereby, it was clear that glycoproteins captured by GNA are involved in a broad range of biological processes such as cell adhesion (GO: 0007155), cellular homeostasis (GO: 0019725), cell communication (GO: 0007154), stress response (GO: 0006950), transmembrane transport (GO: 0055085), etc. However, for specific biological processes relative differences can be found between insects belonging to different orders. For example, the relative amount of glycoproteins associated with transport (GO: 0006810) was 11%, 16%, 15%, 6% and 5% for the glycoproteins derived from *T. castaneum, B. mori*, *A. mellifera*, *D. melanogaster and A. pisum*, respectively. Between the highest and the lowest relative amount of glycoproteins for the category transport (GO: 0006810), a three-fold difference was observed (*A. mellifera* versus *B. mori*). This illustrates a potential differential importance of glycosylation for a particular biological process between insect species belonging to different orders. In addition, it is striking that a large part of the glycoproteins was associated with several metabolic processes.

When glycoproteins were categorized according to molecular function, many of them were involved with hydrolase activity accounting for 19%, 34%, 27%, 31% and 27% in *T. castaneum*, *B. mori, A. mellifera*, *D. melanogaster* and *A. pisum*, respectively. This is in agreement with the many proteases, esterases, glycoside hydrolases, lipases or phosphatases found in the different insect-specific glycoprotein sets (Tables S1, S2, S3, S4, S5). Remarkably, many glycoproteins were also associated with nucleotide binding in the different insects, especially for *A. mellifera* and *A. pisum*. This corresponded with 8%, 11%, 27%, 10% and 15% for *T. castaneum*, *B. mori*, *A. mellifera*, *D. melanogaster* and *A. pisum*, respectively, and correlates with the many ribosomal proteins found in the annotation lists (Tables S1, S2, S3, S4, S5, S6).

## Discussion

One of the major findings in this paper is that very little overlap was observed between the glycoprotein sets derived from the different insect species. This was expected between insect species sampled at different developmental stages (e.g. *Bombyx* larvae and *Tribolium* adults) because glycosylation profiles change depending on reproductive and developmental stage. However, when comparing only adult insects (e.g. *Tribolium* adults and *Drosophila* adults) the diversity in glycoproteins remained extremely high. Since glycosylation is a post-translational modification, changes in carbohydrate composition that were found to be useful during insect evolution can easily be introduced.

Because *N*-glycosylation of proteins occurs in the endoplasmic reticulum (ER) and the Golgi apparatus, it was expected that most glycoproteins would be derived from the luminal part of the secretory pathway such as plasma membrane proteins or secreted proteins. Therefore glycoproteins involved in biological processes such as cell adhesion, cell communication and transmembrane transport were expected to be very dominant. Surprisingly, the cumulative percentage of glycoproteins associated with these processes never exceeded more than 12%. Moreover, it is striking that many glycoproteins were related with metabolic processes associated with certain intracellular compartments such as lysosomes. Many lysosomal enzymes are hydrolases such as proteases, lipases or phosphatases which were found to occur very frequently in the different glycoprotein sets (Tables S1, S2, S3, S4, S5). These enzymes are synthesized by membrane-bound ribosomes on the ER and transverse the ER-Golgi pathway to leave the Golgi apparatus in transport vesicles that fuse with lysosomes. Moreover, in mammalians the presence of mannose-containing *N*-glycans is crucial for lysosomal enzymes to be recognized for trafficking to lysosomes [19]. Recent evidence for a similar lysosomal protein-sorting machinery in *Drosophila* Schneider S2 cells has been found by identifying a homolog of the mammalian mannose 6-phosphate receptor [20]. Our findings support this hypothesis by demonstrating that many enzymes with hydrolytic activities which are known to concentrate in lysosomes contain oligo-mannosidic *N*-glycans.

Another interesting observation was the occurrence of at least 10–25% of (glyco)proteins without a protein signature for the attachment of an *N*-glycan structure. These observations suggest that mannose-containing *O*-glycosylation may be abundantly present in insect species. To our knowledge, the presence of mannose containing *O*-glycans in insects has only been described in *D. melanogaster* for the dystroglycan protein [21], [22]. Moreover, the *O*-mannosyltransferases that are responsible for the *O*-glycosylation were identified as POMT1 and POMT2 [21]. Recessive mutation in a *pomt* gene results in poorly viable flies with defects in muscle development, illustrating the influence of an aberration in *O*-mannosylation on normal development. Using the BLAST search algorithm (EMBL-EBI), we were able to detect predicted protein sequences that are very homologous to POMT1 and POMT2, respectively, for *T. castaneum*, *B. mori, A. mellifera* as well as *A. pisum* (Table S7). The construction of a phylogenetic tree for these predicted POMT proteins revealed that at least two distinct *O*-mannosyltransferases resembling POMT1 and POMT2 are conserved among the five insect species (Figure S2). Many proteins in the different glycoprotein sets have a known cytosolic localization such as actin, tubulin or glycerol-3-phosphate dehydrogenase. Since POMTs are located in the lumen of the Golgi apparatus, cytosolic proteins are not expected to be modified by glycan structures [23]. However, several reports have demonstrated the existence of a cellular system involving retrograde transport of proteins from the ER to the cytosol [24].

A dynamic and abundant *O*-glycosylation of serine and threonine was demonstrated for many cytoplasmic/nuclear proteins [25]–[27]. For example, in *Drosophila*, post-translational *O*-GlcNAc modification was shown to be of importance for the regulation of Polycomb gene expression, while in vertebrates tubulin was even shown to contain sialyloligosaccharides [11], [28], [29]. In addition, other types of cytoplasmic glycosylation may be present. Although at present the expression of a mannosyl transferase in the cytoplasm has never been shown, the addition of mannose residues or mannose containing oligosaccharides to the peptide backbone of cytoplasmic/nuclear proteins may occur in insects.

Apart from its use as a tool for affinity chromatography, the snowdrop (*Galanthus nivalis*) lectin was reported to exert strong insecticidal activity against different insect orders [30]–[32]. Previously, midgut proteins such as ferritin, α-amylase or aminopeptidase were found to be targeted by mannose-binding plant lectins in several economically important pest insects [33]–[36]. Indeed, these three midgut proteins were also found among the GNA binding glycoproteins in several insect species (Table S6). Moreover, this report clearly holds supporting evidence for the hypothesis that plant lectins, and in particular GNA, act on pest insects through the simultaneous interaction with multiple target glycoproteins.

In this manuscript the first comparative study is presented of glycoprotein sets derived from five phylogenetically diverse insect species. Since earlier reports [14]–[17] have shown that the dominant glycan structures in the model insect *D. melanogaster* were of the pauci-mannose *N*-glycan type, the mannose-binding lectin GNA was used in this study to capture insect glycoproteins. However, the percentage of proteins retained on the GNA column was found to be less than 5% of the total protein for the different insect species, suggesting that the number of identified glycoproteins is probably an underestimation of the actual number of glycoproteins. One important reason to explain the low percentage of glycoproteins may be that glycoproteins containing complex glycan structures are more abundant in insects than currently believed, as was recently also shown for *Drosophila* [37]. In addition, the identification of glycoproteins also depends on the quality of the insect databases. As illustrated in Table 1, the number of putative protein sequences present in the different insect databases is highly variable, which may indicate differences in the degree of completion between the insect databases. Subsequently, this will influence protein identification. Therefore, we want to emphasize that the data presented in this report do not intend to give a full database for glycoproteins present in *T. castaneum*, *B. mori*, *A. mellifera*, *D. melanogaster* or *A. pisum*. The glycoprotein catalogs are snapshots of a dynamic glycoproteome during the specific developmental stage of the different insects.

## Materials and Methods

### Insects and lectin purification

All insects were collected from a laboratory colony that was kept at standard conditions. All stages of *T. castaneum* were kept on wheat flour mixed with brewer's yeast (10/1, w/w) [38]. Silkworm *B. mori* (Daizo) larvae were raised on a mulberry-based artificial diet at 25°C (Yakuroto Co., Japan) [39]. After collection from hives of an experimental apiary in Ghent, honeybee workers (*A. mellifera*) were kept at 34°C and 70% relative humidity in laboratory cages and fed with sugar water [40]. A continuous colony of *D. melanogaster* was maintained on a corn meal-based diet, and the pea aphid *A. pisum* was reared on broad beans (*Vicia faba*) at 23–25°C and 65–70% relative humidity [37], [41].

GNA was purified from the bulbs of snowdrop (*Galanthus nivalis*) using a combination of ion exchange chromatography and affinity chromatography [42]. The carbohydrate binding specificity of GNA was previously determined in detail using hapten inhibition assays, frontal affinity chromatography and the glycan array technology provided by the Consortium for Functional Glycomics (http://www.functionalglycomics.org/) [18]. These studies clearly showed that GNA specifically binds to the terminal mannose residues from high-mannose and oligo-mannose *N*-glycans. GNA did not react with more complex *N*-glycans with terminal sugar residues other than mannose.

### Lectin affinity purification of glycoproteins from insect extracts

For the different protein extracts, adult insect bodies were used for the flour beetle *T. castaneum*, the worker honey bee *A. mellifera* and the fruit fly *D. melanogaster*. For the pea aphid *A. pisum* a mix of nymphs and adults was collected, while for the silkworm *B. mori* only fifth larval instar caterpillars were used for extracting proteins. Insect bodies were crushed in liquid nitrogen using a chilled mortar and pestle and an extraction buffer (0.2 M phosphate buffer pH 7.6 containing 2 mM phenylmethanesulfonylfluoride) was added at a ratio of 3 mL buffer per gram of insect powder. The different insect extracts were homogenized using a glass and Teflon homogenizer (10 strokes at 2,000 rpm) and subsequently centrifuged at 9,500 g for 1 h at 4°C. The supernatants were collected and protein concentrations were determined using the Bradford method (Coomassie Protein Assay kit, Thermo scientific, Rockford, IL).

A lectin affinity column (diameter 0.5 cm, height 2 cm) was prepared by coupling the purified GNA to Sepharose 4B using the divinylsulfone method [43]. Approximately 20 mg of total protein was loaded onto the GNA Sepharose column to selectively purify the glycoproteins as described earlier [37]. To circumvent non-specific binding of glycoproteins to GNA, peak fractions were pooled and re-chromatographed on the lectin column. Detailed information on the OD values from the elution fractions of the two subsequent GNA affinity purification steps can be found in Figure S3A–S3E.

To specifically analyze the selectivity of the GNA-affinity column, the binding of several protein extracts was analyzed by SDS-PAGE before and after chemical removal of the glycan structures from the glycoproteins. For the non-specific deglycosylation of the proteins the trifluoromethanesulfonic acid (TFMS) (Sigma-Aldrich) deglycosylation procedure was used [44].

### Preparation of peptides and LC-MS/MS analysis

Glycoproteins eluted from the GNA column were completely dried and re-dissolved in freshly prepared 50 mM ammonium bicarbonate buffer (pH 7.8). Prior to digestion, protein mixtures were boiled for 10 min at 95°C followed by cooling down on ice for 15 min. Sequencing-grade trypsin (Promega, Madison, WI, USA) was added in a 1∶100 (trypsin:substrate) ratio (w/w) and digestion was allowed overnight at 37°C. The sample was acidified with 10% acetic acid (final concentration of 1% acetic acid) and loaded for RP-HPLC separation on a 2.1 mm internal diameter ×150 mm 300SB-C18 column (Zorbax®, Agilent technologies, Waldbronn, Germany) using an Agilent 1100 Series HPLC system. Following a 10 min wash with 10 mM ammonium acetate (pH 5.5) in water/acetonitrile (98/2 (v/v), both Baker HPLC analyzed (Mallinckrodt Baker B.V., Deventer, the Netherlands), a linear gradient to 10 mM ammonium acetate (pH 5.5) in water/acetonitrile (30/70, v/v) was applied over 100 min at a constant flow rate of 80 µL/min. Eluting peptides were collected in 60 fractions between 20 and 80 min, and fractions separated by 15 min were pooled and vacuum dried until further analysis.

These pooled fractions were re-dissolved in 50 µL of 2.5% acetonitrile (HPLC solvent A). Eight µL of this peptide mixture were applied for nanoLC-MS/MS analysis on an Ultimate (Dionex, Amsterdam, the Netherlands) in-line connected to an Esquire HCT mass spectrometer (Bruker, Bremen, Germany). The sample was first trapped on a trapping column (PepMap™ C18 column, 0.3 mm I.D. ×5 mm, Dionex (Amsterdam, the Netherlands)). After back-flushing from the trapping column, the sample was loaded on a 75 µm I.D. ×150 mm reverse-phase column (PepMap™ C18 (Dionex)). The peptides were eluted with a linear gradient of 3% HPLC solvent B (0.1% formic acid in water/acetonitrile (3/7, v/v)) increase per minute at a constant flow rate of 200 nL/min. Using data dependent acquisition multiply charged ions with intensities above threshold (adjusted for each sequence according to the noise level) were selected for fragmentation. During MS/MS analysis, a MS/MS fragmentation amplitude of 0.7 V and a scan time of 40 ms were used.

### Protein identification and bioinformatics

The fragmentation spectra were converted to mgf files using the Automation Engine software (version 3.2, Bruker) and were searched using the MASCOT database search engine (version 2.2.0, Matrix Science, http://www.matrixscience.com) in the appropriate databases. In particular, the Beetlebase (http://www.Beetlebase.org; release Glean.prot.51906), Silkbase (http://silkworm.genomics.org.cn; release Silkworm_glean_pep), Beebase (http://genomes.arc.georgetown.edu; release Amel_pre_release2_OGS_pep), Flybase (http://flybase.org; release FB2010_01) and the Aphidbase (http://www.aphidbase.com/aphidbase; release ACYPproteins) were used to identify proteins from *T. castaneum*, *B. mori*, *A. mellifera*, *D. melanogaster* and *A. pisum*, respectively [45]–[49]. Peptide mass tolerance was set at 0.5 Da and peptide fragment mass tolerance at 0.5 Da, with the ESI-IT as selected instrument for peptide fragmentation rules. Peptide charge was set to 1+,2+,3+. Variable modifications were set to methionine oxidation, pyro-glutamate formation of amino terminal glutamine, acetylation of the N-terminus, deamidation of glutamine or asparagines. The enzyme was set to trypsin. Only peptides that were ranked one and scored above the threshold score set at 95% confidence were withheld. The peptide identification results were made publicly accessible in the proteomics identifications (PRIDE) database (experiment accesion number 13290) (http://www.ebi.ac.uk/pride).

Glycoproteins from different insect species were annotated using the InterProScan tool available from the EBI website (http://www.ebi.ac.uk/Tools/InterProScan) [50]. The InterProScan tool is based on protein databases that use the hidden Markov model methodology to indentify functional protein domains/motives in the primary amino acid sequenes such as Panther, Pfam and TIGR. The obtained IntroProScan output files for *T. castaneum*, *B. mori*, *A. mellifera*, *D. melanogaster* and *A. pisum* can be found in Output File S1, S2, S3, S4, S5. To quantify the presence of certain proteins, an established label-free method was used based on an exponential modified protein abundance index (emPAI) [51], [52]. The emPAI index estimates the abundance of a specific glycoprotein based on the number of identified tryptic peptides. In addition, the number of predicted *N*-glycosylation sites present on the polypeptide backbone was calculated using the NetNGlyc 1.0 server (http://www.cbs.dtu.dk/services/NetNGlyc). Only Asn-X-Ser/Thr sequences (where X is any amino acid except proline) with a prediction score >0.5 were withheld as potential *N*-glycosylation sites. Afterwards the annotated glycoproteins were categorized according to the biological process or molecular function using the Web Gene Ontology Annotation Plot (WEGO) software (http://wego.genomics.org.cn/cgi-bin/wego/index.pl). The WEGO software is a widely used and freely available tool for visualizing, plotting and comparing annotation results based on classification terms provided by the Gene Ontology (GO) Consortium (http://www.geneontology.org/) [53].

## Supporting Information

Figure S1 **Coomassie-stained SDS-PAGE of different elution or run-through fractions obtained after GNA chromatography of protein extracts from *T. castaneum* (T), *D. melanogaster* (D) and *A. pisum* (A).** Lane 0 was loaded with a protein marker (PageRuler™, prestained protein ladder, Fermentas) whereas lanes 1 to 3 were loaded with the peak elution fraction of the GNA chromatography of total proteins extracts from *T. castaneum*, *D. melanogaster* and *A. pisum*, respectively. Lanes 4 to 6 were loaded with run-through samples of GNA chromatography of total protein extracts from *T. castaneum*, *D. melanogaster* and *A. pisum*, respectively. Lanes 7 to 9 were loaded with the peak elution fraction of the GNA chromatography of total proteins extracts after chemical deglycosylation from *T. castaneum*, *D. melanogaster* and *A. pisum*, respectively.
(TIF)

Figure S2 **Phylogenetic tree showing the evolutionary relationship between the homologous protein sequences for** ***O*** **-mannosyltransferase 1 and 2 in** ***D. melanogaster*** **,** ***T. castaneum*** **,** ***B. mori, A. mellifera*** **and** ***A. pisum*** **.**
(TIF)

Figure S3 **Elution profiles of GNA affinity chromatography of total proteins extracts from different insect species.** The eluted fractions from the first chromatography were pooled and rechromatographed on the same GNA column. The OD values of the eluted fractions from the two subsequent GNA affinity chromatography steps from *T. castaneum* (A), *B. mori* (B), *A. mellifera* (C), *D. melanogaster* (D) and *A. pisum* (E) are shown.
(TIF)

Table S1 **Annotation of the identified glycoproteins for** ***Tribolium castaneum*** **.** The list contains the accession number from Beetlebase, an abundance index (emPAI index) and the putative number of *N*-glycosylation sites.
(PDF)

Table S2 **Annotation of the identified glycoproteins for** ***Bombyx mori*** **.** The list contains the accession number from Silkbase, an abundance index (emPAI index) and the putative number of *N*-glycosylation sites.
(PDF)

Table S3 **Annotation of the identified glycoproteins for** ***Apis mellifera*** **.** The list contains the accession number from Beebase, an abundance index (emPAI index) and the putative number of *N*-glycosylation sites.
(PDF)

Table S4 **Annotation of the identified glycoproteins for** ***Drosophila melanogaster*** **.** The list contains the accession number from Flybase, an abundance index (emPAI index) and the putative number of *N*-glycosylation sites.
(PDF)

Table S5 **Annotation of the identified glycoproteins for** ***Acyrthosiphon pisum*** **.** The list contains the accession number from Aphidbase, an abundance index (emPAI index) and the putative number of *N*-glycosylation sites.
(PDF)

Table S6 **Comparative analysis of the number of annotated glycoproteins according to protein description for** ***T. castaneum, B. mori, A. mellifera, D. melanogaster*** **and** ***A. pisum*** **.**
(PDF)

Table S7 **WU-BLAST analysis to search for proteins homologous to** ***O*** **-mannosyltransferases from** ***Drosophila melanogaster*** **POMT1 (Genbank accession No NP_524025.2) and POMT2 (Genbank accession No NP_569858.1).**
(PDF)

Output File S1 **InterProScan output file for *Tribolium castaneum*.**
(OUT)

Output File S2 **InterProScan output file for *Bombyx mori*.**
(OUT)

Output File S3 **InterProScan output file for *Apis mellifera*.**
(OUT)

Output File S4 **InterProScan output file for *Drosophila melanogaster*.**
(OUT)

Output File S5 **InterProScan output file for *Acyrthosiphon pisum*.**
(OUT)
