# Supplementary figures and images for: Diversity in Protein Glycosylation among Insect Species
Source: PLoS One. 2011 Feb 23;6(2):e16682. doi: 10.1371/journal.pone.0016682 (PMC3044136; doi:10.1371/journal.pone.0016682)

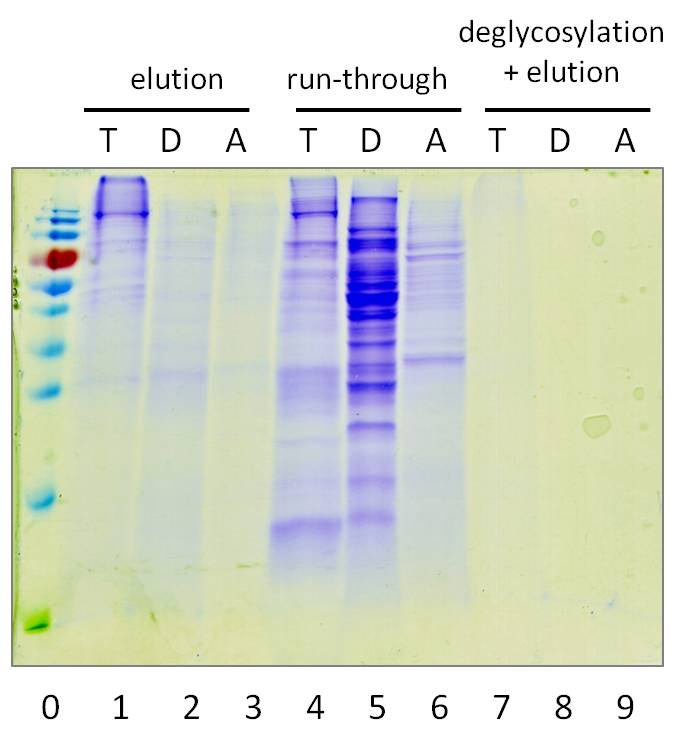

Supplement: Figure S1 — Coomassie-stained SDS-PAGE of different elution or run-through fractions obtained after GNA chromatography of protein extracts from T. castaneum (T), D. melanogaster (D) and A. pisum (A). Lane 0 was loaded with a protein marker (PageRuler™, prestained protein ladder, Fermentas) whereas lanes 1 to 3 were loaded with the peak elution fraction of the GNA chromatography of total proteins extracts from T. castaneum, D. melanogaster and A. pisum, respectively. Lanes 4 to 6 were loaded with run-through samples of GNA chromatography of total protein extracts from T. castaneum, D. melanogaster and A. pisum, respectively. Lanes 7 to 9 were loaded with the peak elution fraction of the GNA chromatography of total proteins extracts after chemical deglycosylation from T. castaneum, D. melanogaster and A. pisum, respectively. (TIF) [file pone.0016682.s001.tif]

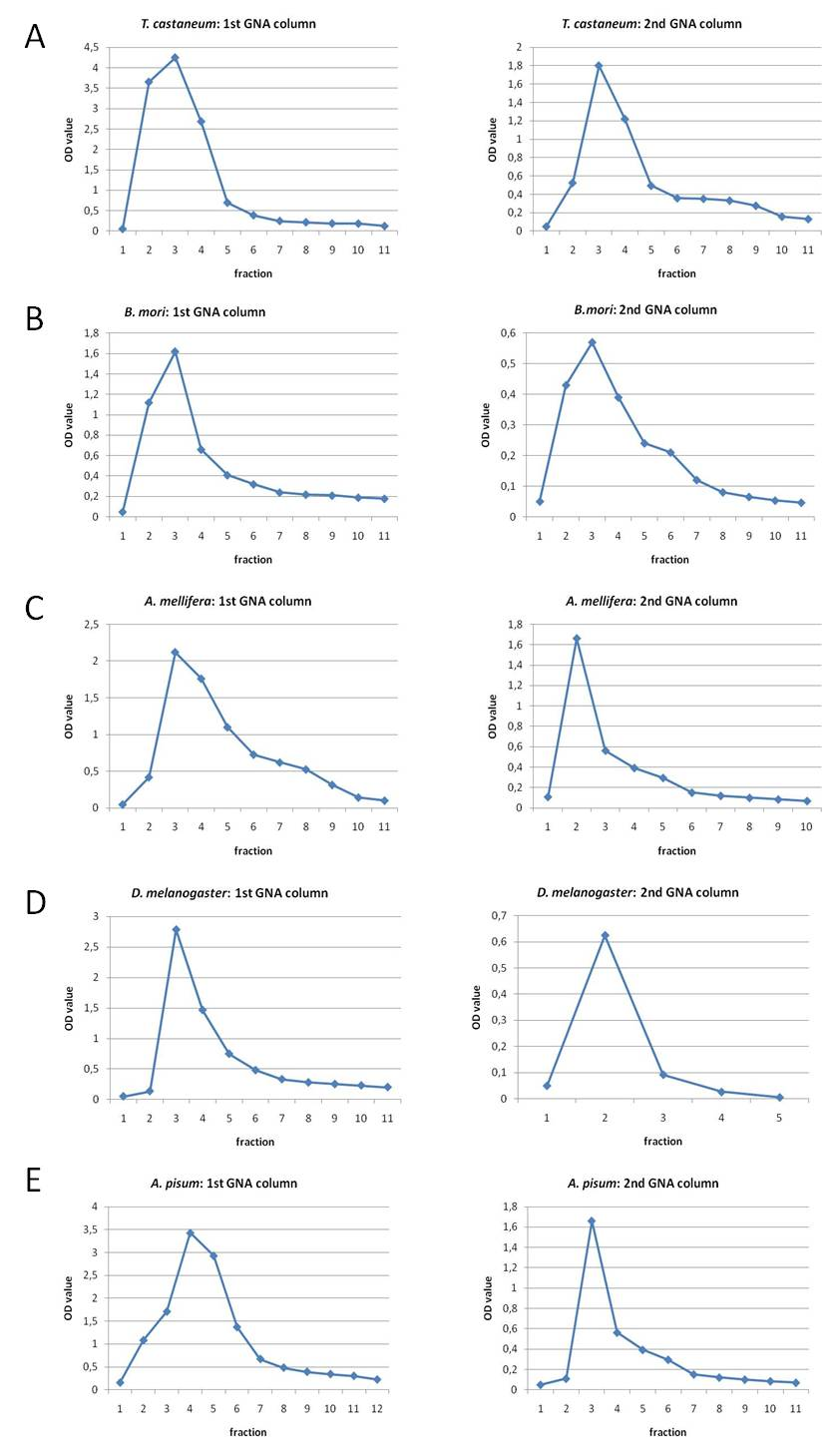

Supplement: Figure S3 — Elution profiles of GNA affinity chromatography of total proteins extracts from different insect species. The eluted fractions from the first chromatography were pooled and rechromatographed on the same GNA column. The OD values of the eluted fractions from the two subsequent GNA affinity chromatography steps from T. castaneum (A), B. mori (B), A. mellifera (C), D. melanogaster (D) and A. pisum (E) are shown. (TIF) [file pone.0016682.s003.tif]
