# Supplementary material for: Diversity in Protein Glycosylation among Insect Species
Source: PLoS One. 2011 Feb 23;6(2):e16682. doi: 10.1371/journal.pone.0016682 (PMC3044136; doi:10.1371/journal.pone.0016682)
Supplement: Table S1 — Annotation of the identified glycoproteins for Tribolium castaneum . The list contains the accession number from Beetlebase, an abundance index (emPAI index) and the putative number of N-glycosylation sites. (PDF) [file pone.0016682.s004.pdf]

Table S1: Annotation of the identified glycoproteins for *Tribolium castaneum* . The list contains the accession number from Beetlebase, an abundance index (emPAI index) and the putative number of N-glycosylation sites.

Protein ID	Protein description	emPAI	putative *N* - glycosylation sites
TC015322	ATP SYNTHASE BETA SUBUNIT	4,0548	1
TC002985	60S RIBOSOMAL PROTEIN L36E	3,6419	0
TC013596	ARGININE KINASE	3,217	0
TC015373	ApoLp-III	1,7823	1
TC013602	VITELLOGENIN-RELATED	1,6736	5
TC003326	ACTIN	1,6369	1
TC010839	VITELLOGENIN-RELATED	1,4288	7
TC003967	60S RIBOSOMAL PROTEIN L14	1,3714	1
TC003672	60S RIBOSOMAL PROTEIN L18	1,1543	0
TC005376	INSECT HEMOCYANIN-RELATED	1,1543	3
TC013536	60S RIBOSOMAL PROTEIN L17	1,1543	0
TC007793	HEAT SHOCK PROTEIN 70KDA	1,0059	4
TC014757	40S RIBOSOMAL PROTEIN S8	0,9953	1
TC010002	60S RIBOSOMAL PROTEIN L12	0,9306	2
TC015161	SHORT-CHAIN DEHYDROGENASE-RELATED	0,8737	0
TC015029-GA	ZINC FINGER PROTEIN GLIS3	0,8548	0
TC008728	ATP SYNTHASE ALPHA SUBUNIT MITOCHONDRIAL	0,848	1
TC014154	INTER-ALPHA-TRYPSIN INHIBITOR HEAVY CHAIN	0,7783	10
TC000476	FERRITIN SUBUNIT 1, INSECT	0,6379	1
TC002464	ALPHA-MANNOSIDASE	0,6125	7
TC006769	INSECT HEMOCYANIN-RELATED	0,6066	3
TC001671	60S RIBOSOMAL PROTEIN L27E	0,5849	0
TC002951	60S RIBOSOMAL PROTEIN L7	0,5849	0
TC005374	INSECT HEMOCYANIN-RELATED	0,4873	2

TC005233	MALIC ENZYME-RELATED	0,4839	4
TC000084-GA	CATALASE	0,4679	2
TC000180	60S RIBOSOMAL PROTEIN L24	0,4679	0
TC002098	60S RIBOSOMAL PROTEIN L35A	0,4679	1
TC003739	HISTONE H1/H5	0,4679	1
TC009596	CYTOCHROME C OXIDASE	0,4679	0
TC013931	ATP SYNTHASE DELTA CHAIN, MITOCHONDRIAL	0,4679	0
TC014183	60S RIBOSOMAL PROTEIN L13	0,4679	1
TC008407	SUSHI DOMAIN CONTAINING PROTEIN	0,425	9
TC005792	60S RIBOSOMAL PROTEIN L11	0,3896	1
TC006106	60S RIBOSOMAL PROTEIN L32	0,3896	0
TC013168	60S RIBOSOMAL PROTEIN L4	0,3896	1
TC015013	60S ACIDIC RIBOSOMAL PROTEIN P2	0,3896	0
TC016306	60S ACIDIC RIBOSOMAL PROTEIN P0	0,3896	0
TC008358	ALPHA-AMYLASE	0,3459	3
TC009485	60S RIBOSOMAL PROTEIN L23	0,3335	1
TC012980	ALANINE AMINOTRANSFERASE	0,3335	0
TC006170	GLYCERALDEHYDE 3-PHOSPHATE DEHYDROGENASE	0,311	2
TC011300	FASCICLIN I	0,311	2
TC015170	3-HYDROXYACYL-COA DEHYROGENASE	0,311	2
TC015539	Ribosomal_S3Ae	0,311	1
TC005232	MALIC ENZYME	0,3011	4
TC001152	HEAT SHOCK PROTEIN 30	0,2915	1
TC002692	SERINE PROTEASE FAMILY S10 SERINE CARBOXYPEPTIDA	0,2915	5
TC005377	INSECT HEMOCYANIN-RELATED	0,2744	3
TC004789	TRANSKETOLASE	0,2589	0
TC008357	ALPHA-AMYLASE	0,2589	2
TC030080	SERINE PROTEASE INHIBITOR, SERPIN	0,2589	0

TC000083	CATALASE	0,2328	1
TC003323	UBIQUINOL-CYTOCHROME C REDUCTASE COMPLEX COR	0,2328	6
TC003496	GLUTATHIONE S-TRANSFERASE	0,2328	0
TC010958	UNCHARACTERIZED	0,2328	1
TC013214	CITRATE SYNTHASE	0,2328	2
TC030666	60S RIBOSOMAL PROTEIN L6	0,2328	0
TC007894	ALPHA-ACTININ	0,2196	0
TC004868	PROTEASOME SUBUNIT ALPHA TYPE 2	0,2114	0
TC004921	CREG1 PROTEIN	0,2114	2
TC006550	RIBOSOMAL PROTEIN S2	0,2114	1
TC008354	V-TYPE ATP SYNTHASE ALPHA CHAIN	0,2114	3
TC008584	PROTEASE FAMILY M28 PLASMA GLUTAMATE CARBOXYI	0,2114	4
TC009602	SERINE PROTEASE-RELATED, INSECT	0,2114	1
TC014574	PROTEASOME SUBUNIT ALPHA TYPE 7	0,2114	1
TC014929	PEROXIREDOXIN	0,2114	0
TC000378	LEUCINE AMINOPEPTIDASE	0,2023	0
TC007349	ARYLSULFATASE B	0,2023	4
TC008246	GLYCOGEN PHOSPHORYLASE	0,2023	5
TC012423	IMMUNOGLOBULIN DOMAIN SUPERFAMILY (SENSORY G	0,1937	4
TC014907	INSECT HEMOCYANIN-RELATED	0,1937	2
TC010489	MULTICOPPER OXIDASE	0,186	1
TC005395	RIBOSOMAL PROTEIN L3P	0,1787	0
TC008261	40S RIBOSOMAL PROTEIN S3	0,1787	1
TC008275	LEUCINE-RICH TRANSMEMBRANE PROTEINS	0,1787	3
TC008684	15-HYDROXYPROSTAGLANDIN DEHYDROGENASE	0,166	2
TC001209	FRUCTOSE-1,6-BISPHOSPHATASE-RELATED	0,1548	1
TC001976	LEUCINE-RICH TRANSMEMBRANE PROTEINS	0,1548	11
TC004566	INORGANIC PYROPHOSPHATASE	0,1548	1

TC009010	ATP SYNTHASE GAMMA SUBUNIT 1	0,1548	2
TC010956	REVERSE TRANSCRIPTASE	0,1513	4
TC011877	UNCHARACTERIZED	0,145	5
TC015655	PUR-TRANSCRIPTIONAL ACTIVATOR	0,145	1
TC006139	MULTIPLE INOSITOL POLYPHOSPHATE PHOSPHATASE	0,1421	4
TC005367	HOMOGENTISATE 1,2-DIOXYGENASE	0,1366	2
TC008729	ACETYL-COA C-ACYLTRANSFERASE	0,1366	2
TC011159	PYRUVATE DEHYDROGENASE E1 COMPONENT, BETA SUE	0,1366	0
TC013570	CADHERIN	0,1345	10
TC004989	CATHEPSIN D	0,1288	2
TC005725	ACONITASE	0,1288	2
TC007948	LEUCINE-RICH TRANSMEMBRANE PROTEIN	0,1288	4
TC001880	ELONGATION FACTOR 1-ALPHA (EF-1-ALPHA)	0,122	1
TC004723	PROTEIN DISULFIDE ISOMERASE	0,122	0
TC005597	DIHYDROLIPOAMIDE ACETYL/SUCCINYL-TRANSFERASE-R	0,122	0
TC007186	LYSOSOMAL ACID LIPASE-RELATED	0,122	1
TC015428	ACYL-COA DEHYDROGENASE	0,122	2
TC002247	SERINE PROTEASE INHIBITOR, SERPIN	0,1158	3
TC004590	14-3-3 protein	0,1158	3
TC011254	SUCCINATE SEMIALDEHYDE DEHYDROGENASE	0,1158	1
TC014998	FRUCTOSE-BISPHOSPHATE ALDOLASE	0,1158	0
TC010540	LAMININ GAMMA-1 CHAIN, INSECT	0,113	9
TC002547	ELECTRON TRANSPORT OXIDOREDUCTASE	0,1105	2
TC005741	SERINE PROTEASE INHIBITOR, SERPIN	0,1105	3
TC009325	FUMARYLACETOACETATE HYDROLASE	0,1105	3
TC003450	PROTEASE S28 PRO-X CARBOXYPEPTIDASE-RELATED	0,1054	2
TC008778	BETA-HEXOSAMINIDASE	0,1054	5
TC009636	ALPHA-GALACTOSIDASE/ALPHA-N-ACETYLGALACTOSAM	0,1054	5

TC016225	PURINE BIOSYNTHESIS PROTEIN 6, PUR6	0,1054	2
TC006555	HEPARAN N-SULFATASE	0,1008	6
TC007253	FASCICLIN II (FAS II)	0,1008	8
TC010864	UNCHARACTERIZED	0,1008	1
TC011207	ENOYL-COA HYDRATASE-RELATED	0,1008	1
TC012702	4-HYDROXYPHENYLPYRUVATE DIOXYGENASE	0,1008	0
TC013056	ASPARTATE AMMONIA LYASE	0,1008	2
TC003279	ADENOSINE DEAMINASE	0,0965	4
TC003230	COLLAGEN ALPHA CHAIN	0,0927	16
TC006189	FAMILY NOT NAMED	0,0927	4
TC015266	CARBOXYLESTERASE	0,0927	2
TC001751	UDP-N-ACTEYLGLUCOSAMINE PYROPHOSPHORYLASE 1	0,0889	0
TC002470	QUIESCIN Q6-RELATED SULFHYDRYL OXIDASE	0,0889	5
TC011039	SPHINGOMYELIN PHOSPHODIESTERASE 1, 2	0,0889	3
TC013193	JUVENILE HORMONE ESTERASE	0,0889	4
TC030624	2-OXOGLUTARATE DEHYDROGENASE	0,0874	3
TC000170	BETA-GALACTOSIDASE RELATED	0,0857	9
TC000188	HEAT SHOCK PROTEIN 70 (HSP70)	0,0857	5
TC000960	ALDEHYDE DEHYDROGENASE	0,0857	1
TC015046	MITOCHONDRIAL PROCESSING PEPTIDASE BETA SUBUNI	0,0857	1
TC007063	UNCHARACTERIZED	0,0827	5
TC015240	ANGIOTENSIN-CONVERTING ENZYME	0,0827	5
TC015262	Periplasmic binding protein-like II	0,0827	5
TC004643	VITELLOGENIN-RELATED	0,0809	0
TC000643	CHAOPTIN	0,0797	8
TC012795	UNCHARACTERIZED	0,0797	5
TC008043	LOW DENSITY LIPOPROTEIN RECEPTOR	0,0784	9
TC013632	CONTACTIN, INSECT	0,0784	9

TC002057	GLUTAMATE DEHYDROGENASE	0,0772	1
TC011019	TROPOMYOSIN INVERTEBRATE	0,0747	4
TC011905	UNCHARACTERIZED	0,0747	7
TC015385	ALDEHYDE DEHYDROGENASE PROTEIN 6	0,0723	4
TC005375	INSECT HEMOCYANIN-RELATED	0,0681	2
TC007868	TBC1 DOMAIN FAMILY MEMBER GTPASE-ACTIVATING PF	0,0681	3
TC011024	HEAT SHOCK PROTEIN 90	0,0681	3
TC014606	HEAT SHOCK PROTEIN 90	0,0681	3
TC013741	VITELLOGENIN-RELATED	0,0661	4
TC001355	UNCHARACTERIZED	0,0578	11
TC003461	LAMININ ALPHA CHAIN, INVERTEBRATE	0,0571	16
TC005184	LAMININ BETA CHAIN-RELATED	0,0524	11
TC013390	PROCOLLAGEN/CELL ADHESION MOLECULE RELATED	0,0524	2
TC002989	ALPHA-L-FUCOSIDASE	0,0512	5
TC012185	HEAT SHOCK PROTEIN 90	0,0512	3
TC000756	LEUCINE-RICH TRANSMEMBRANE PROTEINS	0,0503	18
TC003229	UNCHARACTERIZED	0,0491	19
TC003460	LAMININ ALPHA CHAIN, INVERTEBRATE	0,044	18
TC001262	ALANYL AMINOPEPTIDASE	0,0435	7
TC002089	HEAT SHOCK PROTEIN 70 (HSP70)	0,0428	11
TC005994	NEUROTRACTING	0,0385	14
TC001889	IMMUNOGLOBULIN DOMAIN SUPERFAMILY	0,0349	9
TC014664	UNCHARACTERIZED	0,0299	10
TC013545	UDP-GLUCOSE GLYCOPROTEIN:GLUCOSYLTRANSFERASE	0,0268	2
TC013894	LOW-DENSITY LIPOPROTEIN RECEPTOR	0,0261	27
