# Supplementary material for: Diversity in Protein Glycosylation among Insect Species
Source: PLoS One. 2011 Feb 23;6(2):e16682. doi: 10.1371/journal.pone.0016682 (PMC3044136; doi:10.1371/journal.pone.0016682)
Supplement: Table S2 — Annotation of the identified glycoproteins for Bombyx mori . The list contains the accession number from Silkbase, an abundance index (emPAI index) and the putative number of N-glycosylation sites. (PDF) [file pone.0016682.s005.pdf]

Table S2: Annotation of the identified glycoproteins for *Bombyx mori* . The list contains the accession number from Silkbase, an abundance index (emPAI index) and the putative number of *N* -glycosylation sites.

Protein ID	Protein description	emPAI	putative *N* -glycosylation sites
BGIBMGA002186-PA	PEROXIREDOXIN	1,5119	0
BGIBMGA010979-PA	UNCHARACTERIZED	0,9306	0
BGIBMGA008768-PA	FERRITIN SUBUNIT 1, INSECT	0,5849	1
BGIBMGA004464-PA	Lipoprotein_11	0,5506	1
BGIBMGA007183-PA	SERINE PROTEASE-RELATED	0,4679	0
BGIBMGA009130-PA	40S RIBOSOMAL PROTEIN S20	0,3896	0
BGIBMGA013157-PA	60S ACIDIC RIBOSOMAL PROTEIN P2	0,3896	0
BGIBMGA012788-PA	SERINE PROTEASE-RELATED, INSECT	0,3335	5
BGIBMGA002103-PA	TUBULIN ALPHA CHAIN	0,3183	1
BGIBMGA000353-PA	GRAM-NEGATIVE BACTERIA BINDING PROTEIN 1	0,2915	1
BGIBMGA001280-PA	DEHYDRODOLICHYL DIPHOSPHATE SYNTHASE	0,2915	0
BGIBMGA014404-PA	SERINE PROTEASE-RELATED, INSECT	0,2744	1
BGIBMGA010361-PA	GLYCOGEN PHOSPHORYLASE	0,2497	6
BGIBMGA005576-PA	ACTIN	0,2451	1
BGIBMGA003323-PA	UNCHARACTERIZED	0,2328	0
BGIBMGA005664-PA	ALPHA,ALPHA-TREHALASE	0,2328	5
BGIBMGA013108-PA	Apolipophorin-III	0,2218	0
BGIBMGA001043-PA	60S RIBOSOMAL PROTEIN L27A	0,2114	0
BGIBMGA001201-PA	CALPONIN/TRANSGELIN	0,1937	1
BGIBMGA001584-PA	TROPOMYOSIN INVERTEBRATE	0,1937	1
BGIBMGA001320-PA	SERINE PROTEASE-RELATED, INSECT	0,166	0
BGIBMGA003322-PA	JHBP	0,166	2
BGIBMGA010400-PA	LIPASE	0,166	1
BGIBMGA014429-PA	SERINE PROTEASE-RELATED, INSECT	0,166	0
BGIBMGA011467-PA	60S RIBOSOMAL PROTEIN L18A	0,1548	1

BGIBMGA011844-PA	PROTEIN DISULFIDE ISOMERASE	0,1548	0
BGIBMGA002604-PA	DUF3421	0,145	1
BGIBMGA012831-PA	ALDO-KETO REDUCTASE	0,145	1
BGIBMGA002846-PA	PTD012 PROTEIN	0,1288	2
BGIBMGA007720-PA	SERINE PROTEASE INHIBITOR, SERPIN	0,1288	1
BGIBMGA010867-PA	30S/40S RIBOSOMAL PROTEIN S4	0,1288	1
BGIBMGA013341-PA	MUCIN	0,122	0
BGIBMGA009551-PA	SERINE PROTEASE-RELATED, INSECT	0,1158	2
BGIBMGA010257-PA	SERINE PROTEASE-RELATED, INSECT	0,1054	1
BGIBMGA013945-PA	ACTIN	0,1008	1
BGIBMGA005696-PA	scrB_fam: sucrose-6-phosphate hydrolase	0,0965	3
BGIBMGA002818-PA	DISULFIDE OXIDOREDUCTASE	0,0927	2
BGIBMGA009028-PA	INSECT HEMOCYANIN-RELATED	0,0889	2
BGIBMGA013342-PA	MUCIN	0,0889	2
BGIBMGA010812-PA	GLYCOSIDE HYDROLASES	0,0857	1
BGIBMGA014227-PA	MYOSIN	0,0847	2
BGIBMGA001841-PA	CARBOXYLESTERASE	0,0827	6
BGIBMGA005240-PA	ALPHA-AMYLASE	0,0827	1
BGIBMGA008214-PA	ASPARTATE AMMONIA LYASE	0,0827	2
BGIBMGA009983-PA	RIBOSOME BIOGENESIS PROTEIN BRIX	0,0827	1
BGIBMGA010876-PA	Lipoprotein_11	0,0827	4
BGIBMGA005493-PA	ENOLASE	0,0797	1
BGIBMGA007728-PA	GLUTAMATE CARBOXYPEPTIDASE	0,0747	0
BGIBMGA008164-PA	Lipoprotein_11	0,0747	3
BGIBMGA010536-PA	GLYCOSIDE HYDROLASES	0,0747	3
BGIBMGA002241-PA	V-TYPE ATP SYNTHASE BETA CHAIN	0,0723	3
BGIBMGA008949-PA	FAMILY NOT NAMED	0,0681	7
BGIBMGA012221-PA	TRANSFERRIN	0,0681	7
BGIBMGA001853-PA	ATP SYNTHASE	0,0607	1
BGIBMGA002527-PA	ANGIOTENSIN-CONVERTING ENZYME	0,0593	4

BGIBMGA008295-PA	V-TYPE ATP SYNTHASE ALPHA CHAIN	0,0593	2
BGIBMGA003788-PA	LONG-CHAIN-FATTY-ACID COA LIGASE	0,0578	1
BGIBMGA008063-PA	ALANYL AMINOPEPTIDASE	0,0471	16
BGIBMGA009027-PA	INSECT HEMOCYANIN-RELATED	0,0452	2
BGIBMGA009463-PA	HOST CELL FACTOR-RELATED	0,0404	3
BGIBMGA008551-PA	HEAT SHOCK PROTEIN 70KDA	0,0397	6
BGIBMGA012240-PA	LAMININ	0,0359	10
BGIBMGA014226-PA	MYOSIN	0,0335	9
BGIBMGA007512-PA	UDP-GLUCOSE GLYCOPROTEIN:GLUCOSYLTRANSFERASE	0,0297	0
