# Supplementary material for: Diversity in Protein Glycosylation among Insect Species
Source: PLoS One. 2011 Feb 23;6(2):e16682. doi: 10.1371/journal.pone.0016682 (PMC3044136; doi:10.1371/journal.pone.0016682)
Supplement: Table S3 — Annotation of the identified glycoproteins for Apis mellifera . The list contains the accession number from Beebase, an abundance index (emPAI index) and the putative number of N-glycosylation sites. (PDF) [file pone.0016682.s006.pdf]

Table S3: Annotation of the identified glycoproteins for *Apis mellifera* . The list contains the accession number from Beebase, an abundance index (emPAI index) and the putative number of *N* -glycosylation sites.

Protein ID	Protein description	emPAI	putative *N* - glycosylation sites
GB19422-PA	ISOCITRATE DEHYDROGENASE [NAD]	2,8309	0
GB10973-PA	ARGININE KINASE	2,4554	0
GB13596-PA	ATP SYNTHASE BETA SUBUNIT	1,7214	0
GB10275-PA	TUBULIN BETA CHAIN	1,5119	2
GB14791-PA	ATP SYNTHASE	1,4485	1
GB11613-PA	GLYCEROL-3-PHOSPHATE DEHYDROGENASE	1,1543	0
GB11892-PA	GLYCOGEN PHOSPHORYLASE	0,9829	5
GB12453-PA	ACTIN	0,9683	1
GB11299-PA	60S RIBOSOMAL PROTEIN L19	0,7783	0
GB12140-PA	40S RIBOSOMAL PROTEIN S18	0,7783	0
GB13741-PA	40S RIBOSOMAL PROTEIN S8	0,7783	1
GB18417-PA	ATP SYNTHASE F CHAIN, MITOCHONDRIAL-RELATED	0,7783	0
GB19498-PA	UBIQUITIN-CONJUGATING ENZYME E2	0,7783	0
GB10009-PA	TUBULIN ALPHA CHAIN	0,6237	2
GB10913-PA	40S RIBOSOMAL PROTEIN S17	0,5849	0
GB15291-PA	ATP SYNTHASE GAMMA SUBUNIT 1	0,5849	2
GB17629-PA	60S RIBOSOMAL PROTEIN L8	0,5849	0
GB11028-PA	SPECTRIN-LIKE CELL STRUCTURE PROTEIN	0,5346	0
GB15079-PA	GLUTAMATE DEHYDROGENASE	0,5198	1
GB19082-PA	RIBOSOMAL PROTEIN S2	0,5198	0
GB12614-PA	ACTIN	0,5014	1
GB15619-PA	TRANSKETOLASE	0,4925	0
GB12855-PA	2-OXOGLUTARATE DEHYDROGENASE	0,4815	5
GB13101-PA	transmembrane_regions	0,4679	0
GB14138-PA	GLUTATHIONE PEROXIDASE	0,4679	1
GB16882-PA	ELECTRON TRANSPORT OXIDOREDUCTASE	0,4679	1

GB17499-PA	MITOCHONDRIAL CARRIER PROTEIN RELATED	0,4679	0
GB17238-PA	PYRUVATE DEHYDROGENASE E1 COMPONENT, BETA SUBUNIT	0,425	1
GB18647-PA	PROHIBITIN	0,425	2
GB17876-PA	CATION-TRANSPORTING ATPASE	0,4174	3
GB10989-PA	V-TYPE ATP SYNTHASE ALPHA CHAIN	0,4064	3
GB15049-PA	GLUTAMATE SEMIALDEHYDE DEHYDROGENASE	0,3896	4
GB18619-PA	40S RIBOSOMAL PROTEIN S6	0,3896	3
GB19391-PA	SECRETED SUGAR HYDROLASE	0,3896	0
GB13536-PA	SUCCINYL-COA SYNTHETASE BETA CHAIN	0,3593	4
GB20014-PA	GLYCEROL-3-PHOSPHATE DEHYDROGENASE	0,3593	1
GB17641-PA	ALANINE AMINOTRANSFERASE	0,3502	1
GB10675-PA	ADP-RIBOSYLATION FACTOR, ARF	0,3335	1
GB14758-PA	HEAT SHOCK PROTEIN 90	0,3335	3
GB19724-PA	EUKARYOTIC INITIATION FACTOR 4A	0,3335	1
GB13368-PA	3-HYDROXYACYL-COA DEHYROGENASE	0,311	0
GB10514-PA	TUBULIN ALPHA CHAIN	0,2915	1
GB19208-PA	GTP-BINDING PROTEIN SAR1	0,2915	0
GB20114-PA	RIBOSOMAL PROTEIN L5-RELATED	0,2915	0
GB12573-PA	CITRATE SYNTHASE	0,2744	2
GB12797-PA	TREHALOSE-6-PHOSPHATE SYNTHASE	0,2688	3
GB12338-PA	60S ACIDIC RIBOSOMAL PROTEIN P0	0,2589	0
GB14798-PA	GLYCERALDEHYDE 3-PHOSPHATE DEHYDROGENASE	0,2589	2
GB19380-PA	PEROXIREDOXIN	0,2589	0
GB17113-PA	PHOSPHOFRUCTOKINASE	0,2497	1
GB10590-PA	PHOSPHATIDYLINOSITOL TRANSFER PROTEIN	0,2328	0
GB12951-PA	14-3-3 protein	0,2328	3
GB15355-PA	NADH-UBIQUINONE OXIDOREDUCTASE-RELATED	0,2328	1
GB17038-PA	NADH-UBIQUINONE OXIDOREDUCTASE 24 KDA SUBUNIT	0,2328	0
GB10732-PA	HEAT SHOCK PROTEIN 70KDA	0,2181	8
GB12113-PA	VOLTAGE-DEPENDENT ANION-SELECTIVE CHANNEL	0,2114	5
GB14832-PA	SUCCINATE DEHYDROGENASE IRON-SULFUR PROTEIN	0,2114	2
GB18719-PA	ALCOHOL DEHYDROGENASE RELATED	0,2114	1

GB10560-PA	ELONGATION FACTOR 1-ALPHA	0,1937	0
GB16473-PA	60S RIBOSOMAL PROTEIN L11	0,1937	5
GB16579-PA	ELECTRON TRANSPORT OXIDOREDUCTASE	0,1937	2
GB16742-PA	SARCALUMENIN	0,1937	0
GB11076-PA	RECEPTOR FOR ACTIVATED PROTEIN KINASE C (RACK1)	0,1787	2
GB11385-PA	MITOCHONDRIAL PROCESSING PEPTIDASE BETA SUBUNIT	0,1787	0
GB15172-PA	ASPARTATE AMMONIA LYASE	0,1787	3
GB16464-PA	MALATE AND LACTATE DEHYDROGENASE	0,1787	4
GB16903-PA	CATHEPSIN D	0,1787	1
GB19885-PA	PYRUVATE CARBOXYLASE	0,17	1
GB12182-PA	ACETYL-COA C-ACYLTRANSFERASE	0,166	1
GB16619-PA	NEPRILYSIN	0,166	10
GB11273-PA	RETINOID-INDUCIBLE SERINE CARBOXYPEPTIDASE	0,1548	2
GB11563-PA	MACROGLOBULIN/COMPLEMENT	0,145	1
GB12341-PA	NUCLEAR PROTEIN SKIP-RELATED	0,145	0
GB12383-PA	KETOACID-COENZYME A TRANSFERASE	0,145	2
GB13613-PA	INSECT HEMOCYANIN-RELATED	0,145	2
GB16886-PA	PHOSPHATIDYLCHOLINE-STEROL ACYLTRANSFERASE-RELATED	0,145	3
GB11056-PA	PHOSPHOGLYCERATE KINASE	0,1366	2
GB16443-PA	PYRUVATE DEHYDROGENASE E1 COMPONENT, ALPHA SUBUNIT	0,1366	1
GB19864-PA	CDP-DIACYLGLYCEROL-GLYCEROL-3-PHOSPHATE 3-PHOSPHATIDYLTR	0,1366	0
GB10122-PA	TUBULIN BETA CHAIN	0,122	2
GB14747-PA	HEPARAN N-SULFATASE	0,122	4
GB16600-PA	BETA-HEXOSAMINIDASE	0,122	3
GB18969-PA	CHAPERONIN	0,122	2
GB11973-PA	CYTOCHROME P450 SUBFAMILY 4G	0,1158	1
GB19171-PA	V-TYPE ATP SYNTHASE BETA CHAIN	0,1158	3
GB20080-PA	CHAPERONIN	0,1105	2
GB11810-PA	CONTACTIN, INSECT	0,1054	9
GB16316-PA	ALPHA,ALPHA-TREHALASE	0,1054	6
GB11380-PA	VACUOLAR ATP SYNTHASE SUBUNIT C	0,1008	4
GB15016-PA	HEAT SHOCK PROTEIN 70 (HSP70)	0,1008	0

GB10696-PA	DIHYDROPYRIDINE-SENSITIVE L-TYPE CALCIUM CHANNEL	0,0985	13
GB14947-PA	RIBOPHORIN I	0,0965	3
GB10140-PA	ENOLASE-PHOSPHATASE E-1	0,0927	10
GB14852-PA	HEAT SHOCK PROTEIN 70 (HSP70)	0,0927	6
GB20070-PA	ALPHA-AMYLASE	0,0927	11
GB16429-PA	GLUCOSE-6-PHOSPHATE ISOMERASE	0,0889	4
GB12488-PA	ACONITASE, MITOCHONDRIAL	0,0857	2
GB12693-PA	SOLUTE CARRIER FAMILY 27 (FATTY ACID TRANSPORTER)	0,0857	4
GB13865-PA	PLASMA MEMBRANE CA-ATPASE B2	0,0857	4
GB14361-PA	INSECT HEMOCYANIN-RELATED	0,0827	1
GB14520-PA	FASCICLIN II (FAS II)	0,0747	6
GB13772-PA	PUROMYCIN-SENSITIVE AMINOPEPTIDASE	0,0681	2
GB20055-PA	CATION-TRANSPORTING ATPASE	0,0593	4
GB18010-PA	NITRIC OXIDE SYNTHASE	0,0551	6
GB10992-PA	ATP-CITRATE SYNTHASE	0,0537	7
GB18372-PA	GLYCOGEN DEBRANCHING ENZYME	0,0428	4
GB14634-PA	BETA-HEXOSAMINIDASE	0,0404	12
GB17753-PA	ALANYL AMINOPEPTIDASE	0,0402	20
GB17989-PA	CADHERIN	0,0378	14
GB11059-PA	MUCIN	0,0347	2
GB17654-PA	TRANSMEMBRANE PROTEASE, SERINE 2	0,0344	18
GB18332-PA	UNCHARACTERIZED	0,032	7
GB15133-PA	FAMILY NOT NAMED	0,0271	15
GB12775-PA	LOW-DENSITY LIPOPROTEIN RECEPTOR (LDL)	0,0132	37
GB15664-PA	SPECTRIN BETA CHAIN	0,0132	18
GB17035-PA	MUCIN	0,013	37
