# Supplementary material for: Diversity in Protein Glycosylation among Insect Species
Source: PLoS One. 2011 Feb 23;6(2):e16682. doi: 10.1371/journal.pone.0016682 (PMC3044136; doi:10.1371/journal.pone.0016682)
Supplement: Table S4 — Annotation of the identified glycoproteins for Drosophila melanogaster . The list contains the accession number from Flybase, an abundance index (emPAI index) and the putative number of N-glycosylation sites. (PDF) [file pone.0016682.s007.pdf]

Table S4: Annotation of the identified glycoproteins for *Drosophila melanogaster* . The list contains the accession number from Flybase, an abundance index (emPAI index) and the putative number of *N* -glycosylation sites.

Protein ID	Protein description	emPAI	putative *N* -glycosylation sites
FBpp0071495	PLC-like phosphodiesterases	2,1623	9
FBpp0088250	ATP SYNTHASE BETA SUBUNIT	2,02	0
FBpp0079150	C-TYPE LECTIN DOMAIN CONTAINING PROTEIN	1,6104	3
FBpp0085139	CHAOPTIN	1,3313	12
FBpp0072365	INSECT HEMOCYANIN-RELATED	1,3099	1
FBpp0080795	MACROGLOBULIN/COMPLEMENT	1,2387	12
FBpp0070150	60S RIBOSOMAL PROTEIN L36E	1,1543	0
FBpp0289245	UNCHARACTERIZED	0,8737	3
FBpp0076111	LAMININ	0,8647	9
FBpp0076722	LAMININ ALPHA CHAIN, INVERTEBRATE	0,8412	19
FBpp0079415	C-TYPE LECTIN DOMAIN CONTAINING PROTEIN	0,7783	2
FBpp0076270	ARGININE KINASE	0,7014	0
FBpp0074612	SERINE PROTEASE INHIBITOR, SERPIN	0,6877	2
FBpp0079133	MACROGLOBULIN/COMPLEMENT	0,6066	7
FBpp0071846	40S RIBOSOMAL PROTEIN S24	0,5849	1
FBpp0080043	PROKAR_LIPOPROTEIN	0,5849	5
FBpp0080214	SERINE PROTEASE-RELATED, INSECT	0,5849	1
FBpp0080723	RIBOSOMAL PROTEIN L30	0,5849	0
FBpp0081062	TUBULIN ALPHA CHAIN	0,5198	1
FBpp0079113	LAMININ BETA-1 CHAIN, INSECT	0,4815	9
FBpp0070940	Ribosomal protein L22	0,4679	0
FBpp0075508	CYSTEINE PROTEASE FAMILY C1-RELATED	0,4679	4
FBpp0075735	CARBOXYLESTERASE-6	0,4679	4
FBpp0288658	Crustacean CHH/MIH/GIH neurohormone	0,4679	2
FBpp0082643	VITELLOGENIN-RELATED	0,4405	11
FBpp0083269	SERINE PROTEASE FAMILY S10 SERINE CARBOXYPEPTIDASE	0,4125	0
FBpp0072083	Ribosomal_L11	0,3896	0
FBpp0076359	60S RIBOSOMAL PROTEIN L14	0,3896	0
FBpp0071525	GLYCOGEN DEBRANCHING ENZYME	0,3769	5
FBpp0072177	TUBULIN BETA CHAIN	0,369	3
FBpp0081524	TUBULIN BETA CHAIN	0,369	2
FBpp0070172	UNCHARACTERIZED	0,3593	3
FBpp0070190	UNCHARACTERIZED	0,3593	3
FBpp0086905	DUF3421	0,3593	0

FBpp0071154	Fibronectin type III	0,3406	6
FBpp0074086	40S RIBOSOMAL PROTEIN S19	0,3335	0
FBpp0085717	Ribosomal_L5	0,3335	1
FBpp0083802	40S RIBOSOMAL PROTEIN S3	0,311	1
FBpp0077115	CELL ADHESION MOLECULE	0,3011	9
FBpp0077646	FAMILY NOT NAMED	0,3011	2
FBpp0088252	MUCIN	0,2978	4
FBpp0072355	UNCHARACTERIZED	0,2915	7
FBpp0076602	60S RIBOSOMAL PROTEIN L18	0,2915	0
FBpp0079440	VITELLOGENIN-RELATED	0,2915	0
FBpp0084617	60S RIBOSOMAL PROTEIN L4	0,2744	0
FBpp0082782	PERIOSTIN-RELATED	0,2688	4
FBpp0072973	Ins_allergen_rp	0,2589	1
FBpp0074923	DUF3421	0,2589	2
FBpp0076040	CYSTEINE-RICH SECRETORY PROTEIN	0,2589	7
FBpp0076750	SERINE PROTEASE-RELATED, INSECT	0,2589	0
FBpp0077479	SERINE PROTEASE-RELATED, INSECT	0,2589	3
FBpp0079653	SERINE PROTEASE-RELATED, INSECT	0,2589	1
FBpp0081437	SUCCINYL-COA SYNTHETASE BETA CHAIN	0,2589	0
FBpp0086315	ALPHA-GALACTOSIDASE/ALPHA-N-ACETYLGALACTOSAMINIDASE	0,2589	2
FBpp0099686	40S RIBOSOMAL PROTEIN S8	0,2589	1
FBpp0078557	CONTACTIN, INSECT	0,2549	10
FBpp0087506	PHOSPHOFRUCTOKINASE	0,252	2
FBpp0070633	Fibronectin type III	0,2388	7
FBpp0071075	UNCHARACTERIZED	0,2328	2
FBpp0076335	SERINE PROTEASE-RELATED, INSECT	0,2328	0
FBpp0077261	PROKAR_LIPOPROTEIN	0,2328	3
FBpp0078677	RETICULON-RELATED	0,2328	0
FBpp0079321	C-TYPE LECTIN DOMAIN CONTAINING PROTEIN	0,2328	2
FBpp0079500	40S RIBOSOMAL PROTEIN S2	0,2328	0
FBpp0078982	CRUMBS(D.MELANOGASTER)-RELATED	0,2274	23
FBpp0077109	Fibronectin type III	0,2218	7
FBpp0084630	TUBULIN BETA CHAIN	0,2218	4
FBpp0071185	UNCHARACTERIZED	0,2114	5
FBpp0078690	SERINE PROTEASE-RELATED, INSECT	0,2114	0
FBpp0087559	GALACTOSE-SPECIFIC C-TYPE LECTIN	0,2114	1
FBpp0088242	40S RIBOSOMAL PROTEIN S3A	0,2114	1
FBpp0100044	ALPHA-L-FUCOSIDASE	0,2114	2
FBpp0271712	FIBRINOGEN-RELATED	0,2114	3
FBpp0076608	PROTEASE S28 PRO-X CARBOXYPEPTIDASE-RELATED	0,2023	3

FBpp0071343	Fibrinogen_C	0,1937	2
FBpp0079320	C-TYPE LECTIN DOMAIN CONTAINING PROTEIN	0,1937	1
FBpp0083548	THIOREDOXIN_2	0,1937	4
FBpp0085928	ZN/RING FINGER PROTEIN 2	0,1937	3
FBpp0071794	ATP SYNTHASE ALPHA SUBUNIT MITOCHONDRIAL	0,186	0
FBpp0085915	RNA-BINDING PROTEIN	0,1722	3
FBpp0081114	GLUCOSE DEHYDROGENASE	0,166	3
FBpp0083855	UNCHARACTERIZED	0,166	4
FBpp0079297	ANGIOTENSIN-CONVERTING ENZYME	0,1601	4
FBpp0074167	LIPASE	0,1548	2
FBpp0070041	Protein kinase-like (PK-like)	0,145	2
FBpp0070890	26S PROTEASE REGULATORY SUBUNIT S10B	0,145	1
FBpp0082494	GLYCOGEN SYNTHASE	0,145	1
FBpp0112535	SERINE PROTEASE-RELATED, INSECT	0,145	5
FBpp0074549	ISOCITRATE DEHYDROGENASE	0,1366	0
FBpp0077536	ASPARTATE AMINOTRANSFERASE	0,1366	1
FBpp0070435	LAMININ	0,1316	0
FBpp0070787	Actin-LIKE	0,1288	1
FBpp0079187	RECEPTOR FOR ACTIVATED PROTEIN KINASE C	0,1288	1
FBpp0084275	ISOCITRATE DEHYDROGENASE [NAD]	0,122	0
FBpp0080895	PROLYLCARBOXYPEPTIDASE/PRCP	0,1158	6
FBpp0081110	PROTEIN DISULFIDE ISOMERASE	0,1158	4
FBpp0079406	ALDEHYDE DEHYDROGENASE	0,1105	2
FBpp0083524	PROTEASE M1 ZINC METALLOPROTEASE	0,1076	10
FBpp0079101	Terpenoid cyclases/Protein prenyltransferases	0,1054	8
FBpp0071733	CELL ADHESION MOLECULE	0,1008	3
FBpp0078603	BETA-MANNOSIDASE	0,1008	10
FBpp0083526	ALANYL AMINOPEPTIDASE	0,1008	12
FBpp0079621	ALPHA-MANNOSIDASE	0,0985	4
FBpp0076310	GLYPICAN	0,0965	3
FBpp0073263	ACID PHOSPHATASE RELATED	0,0927	5
FBpp0082248	ACETYLCHOLINESTERASE	0,0889	3
FBpp0080129	ANGIOTENSIN-CONVERTING ENZYME	0,0857	3
FBpp0085777	BETA-GALACTOSIDASE	0,0827	5
FBpp0099974	SERINE/THREONINE PROTEIN PHOSPHATASE	0,0827	1
FBpp0071192	BETA-HEXOSAMINIDASE	0,0797	4
FBpp0081089	Fibronectin type III	0,0797	11
FBpp0073523	ZINC METALLOPROTEASE	0,0772	6
FBpp0085736	SUCCINATE DEHYDROGENASE 2 FLAVOPROTEIN SUBUNIT	0,0747	0
FBpp0077168	NICOTINATE PHOSPHORIBOSYLTRANSFERASE	0,07	5

FBpp0084624	ZINC METALLOPROTEASE	0,07	8
FBpp0271723	DOMON	0,0681	6
FBpp0078531	ZINC METALLOPROTEASE FAMILY M13 NEPRILYSIN-RELATED	0,0661	8
FBpp0079453	3-HYDROXYACYL-COA DEHYROGENASE	0,0661	1
FBpp0076507	LEUCINE-RICH TRANSMEMBRANE PROTEIN	0,0641	10
FBpp0082118	Creatinase/aminopeptidase	0,0641	3
FBpp0084718	ZINC METALLOPROTEASE	0,0624	4
FBpp0084772	AMINOPEPTIDASE N-RELATED	0,0624	5
FBpp0084663	BPTI_KUNITZ_2	0,0619	11
FBpp0086050	SEMAPHORIN	0,0607	6
FBpp0081963	MIDLINE FASCICLIN	0,0593	6
FBpp0083401	UNCHARACTERIZED	0,0551	11
FBpp0084770	AMINOPEPTIDASE N-RELATED	0,0551	4
FBpp0077121	TREHALOSE-6-PHOSPHATE SYNTHASE	0,0537	1
FBpp0079632	ALPHA-MANNOSIDASE	0,0537	9
FBpp0073966	Clathrin	0,0512	7
FBpp0083523	ALANYL AMINOPEPTIDASE	0,0503	11
FBpp0084767	AMINOPEPTIDASE N-RELATED	0,0481	4
FBpp0075023	2-OXOGLUTARATE DEHYDROGENASE	0,0471	3
FBpp0077154	Fibronectin type III	0,0471	6
FBpp0071853	EGF	0,0404	7
FBpp0111856	DIHYDROPYRIDINE-SENSITIVE L-TYPE CALCIUM CHANNEL	0,0404	7
FBpp0087438	LOW DENSITY LIPOPROTEIN RECEPTOR	0,0378	5
FBpp0075730	NEUREXIN IV	0,0361	7
FBpp0075731	Galactose-binding domain-like	0,0356	8
FBpp0075645	PROTEIN TYROSINE PHOSPHATASE 69D, DROME	0,0299	22
FBpp0079064	MYOSIN	0,0287	5
FBpp0070200	Uncharacterized	0,0228	18
