# Supplementary material for: Diversity in Protein Glycosylation among Insect Species
Source: PLoS One. 2011 Feb 23;6(2):e16682. doi: 10.1371/journal.pone.0016682 (PMC3044136; doi:10.1371/journal.pone.0016682)
Supplement: Table S5 — Annotation of the identified glycoproteins for Acyrthosiphon pisum . The list contains the accession number from Aphidbase, an abundance index (emPAI index) and the putative number of N-glycosylation sites. (PDF) [file pone.0016682.s008.pdf]

**Table S5:** Annotation of the identified glycoproteins for *Acyrthosiphon pisum* . The list contains the accession number from Aphidbase, an abundance index (emPAI index) and the putative number of *N* -glycosylation sites.

| Protein ID | Protein description | emPAI | putative *N* - glycosylation sites |
|---|---|---|---|
| ACYPI009769-PA | GLYCERALDEHYDE 3-PHOSPHATE DEHYDROGENASE | 3,6419 | 2 |
| ACYPI005249-PA | UNCHARACTERIZED | 2,8309 | 0 |
| ACYPI002286-PA | AAA-FAMILY ATPASE | 2,5286 | 2 |
| ACYPI000011-PA | SERINE CARBOXYPEPTIDASE | 2,3404 | 5 |
| ACYPI008874-PA | TUBULIN BETA CHAIN | 2,3243 | 2 |
| ACYPI47197-PA | CHAPERONIN | 2,1623 | 0 |
| ACYPI004403-PA | AMINOPEPTIDASE N-RELATED | 1,4848 | 6 |
| ACYPI001736-PA | UNCHARACTERIZED | 1,4712 | 5 |
| ACYPI23515-PA | LEUCINE-RICH TRANSMEMBRANE PROTEINS | 1,2537 | 5 |
| ACYPI000064-PA | ACTIN | 1,2387 | 1 |
| ACYPI34982-PA | LEUCINE-RICH TRANSMEMBRANE PROTEINS | 1,2387 | 5 |
| ACYPI000071-PA | 40S RIBOSOMAL PROTEIN S15 | 1,1543 | 0 |
| ACYPI008976-PA | ACID PHOSPHATASE-1 | 1,1543 | 5 |
| ACYPI48450-PA | LEUCINE-RICH TRANSMEMBRANE PROTEIN | 1,1543 | 3 |
| ACYPI004741-PA | GLUCOSE DEHYDROGENASE | 1,0431 | 2 |
| ACYPI004906-PA | LEUCINE-RICH TRANSMEMBRANE PROTEIN | 1,0091 | 8 |
| ACYPI001302-PA | ALPHA-GALACTOSIDASE/ALPHA-N-ACETYLGALACTOSAMINIDASE | 0,9055 | 6 |
| ACYPI008776-PA | CLATHRIN HEAVY CHAIN | 0,878 | 7 |
| ACYPI009513-PA | ALPHA-GALACTOSIDASE/ALPHA-N-ACETYLGALACTOSAMINIDASE | 0,8587 | 3 |
| ACYPI006238-PA | EUKARYOTIC TRANSLATION INITIATION FACTOR 3F, EIF3F | 0,848 | 1 |
| ACYPI001776-PA | LAMININ | 0,8382 | 9 |
| ACYPI003702-PA | PROCOLLAGEN/CELL ADHESION MOLECULE RELATED | 0,8022 | 4 |
| ACYPI001315-PA | CELL ADHESION MOLECULE | 0,7783 | 6 |
| ACYPI001436-PA | ALPHA-AMYLASE | 0,7783 | 3 |
| ACYPI002010-PA | HEAT SHOCK PROTEIN 90 | 0,7783 | 4 |
| ACYPI003547-PA | MACROPHAGE MIGRATION INHIBITORY FACTOR RELATED | 0,7783 | 1 |
| ACYPI004434-PA | ELONGATION FACTOR 1-ALPHA | 0,7783 | 1 |

| | | | |
|---|---|---|---|
| ACYPI006035-PA | ACTIN | 0,7783 | 0 |
| ACYPI008325-PA | 40S RIBOSOMAL PROTEIN S2 | 0,7783 | 0 |
| ACYPI009454-PA | 40S RIBOSOMAL PROTEIN S24 | 0,7783 | 1 |
| ACYPI007079-PA | PROLYL 4-HYDROXYLASE ALPHA SUBUNIT | 0,7474 | 4 |
| ACYPI23892-PA | VITELLOGENIN-RELATED | 0,7342 | 8 |
| ACYPI000580-PA | NEPRILYSIN | 0,7084 | 6 |
| ACYPI001461-PA | GLUTAMINE SYNTHETASE | 0,7014 | 2 |
| ACYPI004676-PA | GALACTOSE-SPECIFIC C-TYPE LECTIN | 0,7014 | 0 |
| ACYPI21131-PA | LEUCINE-RICH TRANSMEMBRANE PROTEIN | 0,7014 | 6 |
| ACYPI32283-PA | LEUCINE-RICH TRANSMEMBRANE PROTEIN | 0,668 | 3 |
| ACYPI002483-PA | EUKARYOTIC INITIATION FACTOR 4A | 0,6497 | 2 |
| ACYPI008065-PA | FATTY ACID SYNTHASE | 0,6237 | 6 |
| ACYPI008926-PA | PROTEIN DISULFIDE ISOMERASE | 0,6237 | 0 |
| ACYPI000091-PA | 40S RIBOSOMAL PROTEIN S26 | 0,5849 | 1 |
| ACYPI000918-PA | LYSOSOMAL ALPHA-MANNOSIDASE | 0,5849 | 7 |
| ACYPI001447-PA | CARBOXYLESTERASE | 0,5849 | 3 |
| ACYPI003079-PA | DEOXYRIBONUCLEASE II | 0,5849 | 3 |
| ACYPI008181-PA | 60S RIBOSOMAL PROTEIN L17 | 0,5849 | 0 |
| ACYPI30377-PA | LEUCINE-RICH TRANSMEMBRANE PROTEINS | 0,5849 | 1 |
| ACYPI000371-PA | LYSOSOMAL ALPHA-MANNOSIDASE | 0,5707 | 11 |
| ACYPI007662-PA | LAMININ | 0,5631 | 11 |
| ACYPI55286-PA | LAMININ | 0,557 | 7 |
| ACYPI000731-PA | LEUCINE-RICH TRANSMEMBRANE PROTEINS | 0,546 | 9 |
| ACYPI000023-PA | EUKARYOTIC TRANSLATION INITIATION FACTOR 3 SUBUNIT 11 | 0,5198 | 0 |
| ACYPI001013-PA | C-type lectin-like | 0,5198 | 2 |
| ACYPI009480-PA | ORNITHINE AMINOTRANSFERASE | 0,5198 | 2 |
| ACYPI003061-PA | YELLOW PROTEIN-RELATED | 0,4925 | 3 |
| ACYPI000039-PA | 60S RIBOSOMAL PROTEIN L30 | 0,4679 | 1 |
| ACYPI000040-PA | 60S ACIDIC RIBOSOMAL PROTEIN P2 | 0,4679 | 1 |
| ACYPI001221-PA | UDP-N-ACETYLGLUCOSAMINE--PEPTIDE N-ACETYLGLUCOSAMINYLTRANSF | 0,4679 | 2 |
| ACYPI004925-PA | ALPHA-GALACTOSIDASE/ALPHA-N-ACETYLGALACTOSAMINIDASE | 0,4679 | 4 |

| | | | |
|---|---|---|---|
| ACYPI006593-PA | LOW DENSITY LIPOPROTEIN RECEPTOR | 0,4679 | 5 |
| ACYPI001790-PA | SERINE PROTEASE INHIBITOR, SERPIN | 0,4454 | 12 |
| ACYPI004819-PA | PROTEASE S28 PRO-X CARBOXYPEPTIDASE-RELATED | 0,4454 | 6 |
| ACYPI006716-PA | PROLYLCARBOXYPEPTIDASE | 0,425 | 6 |
| ACYPI002758-PA | SERINE/THREONINE PROTEIN PHOSPHATASE | 0,4031 | 1 |
| ACYPI000031-PA | 40S RIBOSOMAL PROTEIN S12 | 0,3896 | 2 |
| ACYPI002182-PA | HISTONE H2B | 0,3896 | 0 |
| ACYPI008145-PA | PROTEASE FAMILY C26 GAMMA-GLUTAMYL HYDROLASE | 0,3896 | 3 |
| ACYPI009342-PA | GLYCOGEN DEBRANCHING ENZYME | 0,3896 | 0 |
| ACYPI010127-PA | 40S RIBOSOMAL PROTEIN S3A | 0,3896 | 1 |
| ACYPI49697-PA | UNCHARACTERIZED | 0,3896 | 3 |
| ACYPI003972-PA | PROTEASE S28 PRO-X CARBOXYPEPTIDASE-RELATED | 0,3737 | 4 |
| ACYPI002298-PA | TREHALASE | 0,3593 | 4 |
| ACYPI004314-PA | 26S PROTEASOME NON-ATPASE REGULATORY SUBUNIT 8 | 0,3593 | 2 |
| ACYPI000016-PA | HSP40, SUBFAMILY A, MEMBERS 1,2,4 | 0,3335 | 0 |
| ACYPI000292-PA | LEUCINE-RICH TRANSMEMBRANE PROTEINS | 0,3335 | 4 |
| ACYPI006346-PA | UNCHARACTERIZED | 0,3335 | 0 |
| ACYPI006761-PA | TUBULIN ALPHA CHAIN | 0,3335 | 1 |
| ACYPI009718-PA | 26S PROTESOME SUBUNIT 6 | 0,3335 | 0 |
| ACYPI010073-PA | TUBULIN ALPHA CHAIN | 0,3335 | 1 |
| ACYPI49696-PA | UNCHARACTERIZED | 0,3183 | 1 |
| ACYPI000012-PA | CATHEPSIN B | 0,311 | 2 |
| ACYPI000432-PA | OXIDASE/PEROXIDASE | 0,311 | 7 |
| ACYPI000001-PA | PROTEASE M1 ZINC METALLOPROTEASE | 0,2978 | 14 |
| ACYPI008675-PA | METALLOPROTEASE | 0,2978 | 3 |
| ACYPI000058-PA | COFILIN-RELATED | 0,2915 | 1 |
| ACYPI002498-PA | AMINOPEPTIDASE N-RELATED | 0,2915 | 1 |
| ACYPI002553-PA | 40S RIBOSOMAL PROTEIN S10 | 0,2915 | 0 |
| ACYPI003298-PA | 40S RIBOSOMAL PROTEIN S6 | 0,2915 | 2 |
| ACYPI008080-PA | TCTPROTEIN | 0,2915 | 1 |
| ACYPI003145-PA | CARBOXYLESTERASE | 0,2826 | 4 |

| | | | |
|---|---|---|---|
| ACYPI000061-PA | ATP SYNTHASE BETA SUBUNIT | 0,2688 | 0 |
| ACYPI009732-PA | BETA-GALACTOSIDASE RELATED | 0,2688 | 9 |
| ACYPI003206-PA | EGF-LIKE DOMAIN PROTEIN | 0,2607 | 23 |
| ACYPI000002-PA | ALPHA-AMYLASE | 0,2589 | 3 |
| ACYPI001928-PA | UNCHARACTERIZED | 0,2589 | 3 |
| ACYPI002881-PA | ALPHA-GALACTOSIDASE/ALPHA-N-ACETYLGALACTOSAMINIDASE | 0,2589 | 3 |
| ACYPI006264-PA | BETA-GALACTOSIDASE | 0,2589 | 3 |
| ACYPI009816-PA | 26S PROTEASOME REGULATORY SUBUNIT S3 | 0,2589 | 1 |
| ACYPI009856-PA | RIBOSOMAL PROTEIN S9 | 0,2589 | 0 |
| ACYPI010019-PA | LAMININ | 0,2589 | 11 |
| ACYPI30879-PA | LEUCINE-RICH TRANSMEMBRANE PROTEIN | 0,2589 | 10 |
| ACYPI48447-PA | LEUCINE-RICH TRANSMEMBRANE PROTEIN | 0,2589 | 7 |
| ACYPI008178-PA | MYOSIN | 0,2511 | 8 |
| ACYPI003807-PA | SERINE PROTEASE-RELATED | 0,2451 | 2 |
| ACYPI007027-PA | FRUCTOSE-BISPHOSPHATE ALDOLASE | 0,2451 | 0 |
| ACYPI004352-PA | BETA-HEXOSAMINIDASE | 0,2411 | 5 |
| ACYPI009980-PA | BETA-HEXOSAMINIDASE | 0,2411 | 6 |
| ACYPI000081-PA | 40S RIBOSOMAL PROTEIN S18 | 0,2328 | 0 |
| ACYPI002457-PA | LEUCINE-RICH TRANSMEMBRANE PROTEIN | 0,2328 | 3 |
| ACYPI002825-PA | VON WILLEBRAND FACTOR, TYPE A DOMAIN CONTAINING | 0,2328 | 8 |
| ACYPI004023-PA | MITOCHONDRIAL CARRIER PROTEIN | 0,2328 | 1 |
| ACYPI006814-PA | UNCHARACTERIZED | 0,2218 | 7 |
| ACYPI008897-PA | SERINE PROTEASE INHIBITOR, SERPIN | 0,2218 | 3 |
| ACYPI004271-PA | RIBOSOMAL PROTEIN S2 | 0,2114 | 1 |
| ACYPI007447-PA | Galactose-binding domain-like | 0,2114 | 3 |
| ACYPI008041-PA | PROCOLLAGEN/CELL ADHESION MOLECULE RELATED | 0,2114 | 0 |
| ACYPI009057-PA | TUBULIN | 0,2114 | 1 |
| ACYPI008184-PA | CELL ADHESION MOLECULE | 0,2078 | 9 |
| ACYPI002661-PA | EUKARYOTIC TRANSLATION INITIATION FACTOR 3 SUBUNIT 6-RELATED | 0,2023 | 2 |
| ACYPI004360-PA | INOSINE-5-MONOPHOSPHATE DEHYDROGENASE | 0,2023 | 1 |
| ACYPI000969-PA | TROPOMYOSIN | 0,1937 | 1 |

| | | | |
|---|---|---|---|
| ACYPI002402-PA | ALPHA-GALACTOSIDASE/ALPHA-N-ACETYLGALACTOSAMINIDASE | 0,1937 | 3 |
| ACYPI002662-PA | TRANSKETOLASE | 0,1937 | 1 |
| ACYPI007003-PA | CREG1 PROTEIN | 0,1937 | 1 |
| ACYPI007933-PA | UNCHARACTERIZED | 0,1937 | 2 |
| ACYPI009967-PA | UNCHARACTERIZED | 0,1937 | 4 |
| ACYPI004258-PA | FATTY ACID SYNTHASE | 0,1918 | 8 |
| ACYPI000088-PA | L-XYLULOSE REDUCTASE | 0,1787 | 2 |
| ACYPI001648-PA | FAMILY NOT NAMED | 0,1787 | 1 |
| ACYPI003593-PA | RIBOSOMAL PROTEIN L5 | 0,1787 | 0 |
| ACYPI004024-PA | RAS-RELATED GTPASE | 0,1787 | 0 |
| ACYPI005092-PA | 60S RIBOSOMAL PROTEIN L6 | 0,1787 | 0 |
| ACYPI010069-PA | UNCHARACTERIZED | 0,1787 | 2 |
| ACYPI000588-PA | CHAPERONIN | 0,1722 | 1 |
| ACYPI000079-PA | 60S ACIDIC RIBOSOMAL PROTEIN FAMILY MEMBER | 0,166 | 1 |
| ACYPI000189-PA | JHBP | 0,166 | 4 |
| ACYPI000257-PA | CHAPERONIN | 0,166 | 3 |
| ACYPI001186-PA | 26S PROTEASOME REGULATORY ATPASE SUBUNIT | 0,166 | 0 |
| ACYPI002133-PA | 26S PROTEASOME NON-ATPASE REGULATORY SUBUNIT 4 | 0,166 | 0 |
| ACYPI002440-PA | PROTEASE FAMILY C26 GAMMA-GLUTAMYL HYDROLASE | 0,166 | 5 |
| ACYPI007515-PA | EXTRACELLULAR MATRIX GLYCOPROTEIN RELATED | 0,166 | 5 |
| ACYPI005230-PA | PROTEASE T2 ASPARAGINASE | 0,1548 | 1 |
| ACYPI005376-PA | SECRETED GLUCOSIDASE-RELATED | 0,1548 | 1 |
| ACYPI008059-PA | ALPHA-GLUCOSIDASE | 0,1548 | 7 |
| ACYPI007706-PA | CHAPERONIN | 0,1497 | 2 |
| ACYPI000100-PA | 30S/40S RIBOSOMAL PROTEIN S4 | 0,145 | 1 |
| ACYPI006239-PA | GLUTAMINE SYNTHETASE | 0,145 | 1 |
| ACYPI010112-PA | 26S PROTEASOME REGULATORY SUBUNIT | 0,145 | 2 |
| ACYPI006675-PA | PROTEASE M1 ZINC METALLOPROTEASE | 0,1421 | 7 |
| ACYPI005215-PA | L-ASPARAGINASE | 0,1405 | 8 |
| ACYPI002536-PA | CADHERIN | 0,1345 | 12 |
| ACYPI009151-PA | CHAPERONIN | 0,1327 | 1 |

| | | | |
|---|---|---|---|
| ACYPI004339-PA | EGF-LIKE DOMAIN PROTEIN | 0,1314 | 10 |
| ACYPI005469-PA | ACETYL-COA ACETYLTRANSFERASE | 0,1288 | 1 |
| ACYPI007383-PA | BETA-HEXOSAMINIDASE | 0,1288 | 2 |
| ACYPI007679-PA | 26S PROTEASOME REGULATORY SUBUNIT 7, PSD7 | 0,1288 | 2 |
| ACYPI008920-PA | SERINE CARBOXYPEPTIDASE | 0,1288 | 4 |
| ACYPI009633-PA | EUKARYOTIC TRANSLATION INITIATION FACTOR 3 SUBUNIT 3 | 0,1288 | 1 |
| ACYPI001732-PA | DENDRITIC CELL PROTEIN GA17 | 0,122 | 4 |
| ACYPI005498-PA | GLYPICAN | 0,122 | 1 |
| ACYPI33248-PA | LEUCINE-RICH TRANSMEMBRANE PROTEINS | 0,122 | 6 |
| ACYPI000554-PA | CELL ADHESION MOLECULE | 0,1105 | 6 |
| ACYPI000631-PA | CARBOXYLESTERASE | 0,1105 | 3 |
| ACYPI007621-PA | UNCHARACTERIZED | 0,1105 | 10 |
| ACYPI008775-PA | SERINE PROTEASE INHIBITOR, SERPIN | 0,1105 | 2 |
| ACYPI000180-PA | SERINE PROTEASE INHIBITOR, SERPIN | 0,1076 | 5 |
| ACYPI008996-PA | HEAT SHOCK PROTEIN 70KDA | 0,1076 | 6 |
| ACYPI009915-PA | HEAT SHOCK PROTEIN 90 | 0,1076 | 2 |
| ACYPI000933-PA | 26S PROTEASOME SUBUNIT S9 | 0,1054 | 1 |
| ACYPI005134-PA | ACID PHOSPHATASE-RELATED | 0,1054 | 5 |
| ACYPI006221-PA | LEUCINE-RICH TRANSMEMBRANE PROTEIN | 0,1054 | 7 |
| ACYPI000119-PA | PROTEIN DISULFIDE ISOMERASE | 0,1008 | 1 |
| ACYPI001203-PA | AMINOPEPTIDASE N-RELATED | 0,1008 | 12 |
| ACYPI001272-PA | NADP-SPECIFIC ISOCITRATE DEHYDROGENASE | 0,1008 | 1 |
| ACYPI001622-PA | COATOMER GAMMA SUBUNIT | 0,1008 | 3 |
| ACYPI004825-PA | ANGIOPOIETIN 1 RECEPTOR | 0,1008 | 7 |
| ACYPI006821-PA | 26S PROTEASE REGULATORY SUBUNIT 6B | 0,1008 | 1 |
| ACYPI010227-PA | PROTEASE FAMILY M28 PLASMA GLUTAMATE CARBOXYPEPTIDASE-RELAT | 0,1008 | 2 |
| ACYPI001125-PA | GLYCOGEN PHOSPHORYLASE | 0,0985 | 5 |
| ACYPI003124-PA | DEAD BOX ATP-DEPENDENT RNA HELICASE | 0,0965 | 1 |
| ACYPI004078-PA | PROTEASOME REGULATORY SUBUNITS | 0,0965 | 2 |
| ACYPI009317-PA | LAMININ | 0,0932 | 11 |
| ACYPI003954-PA | CYSTEINE PROTEASE FAMILY C1-RELATED | 0,0927 | 4 |

| | | | |
|---|---|---|---|
| ACYPI005091-PA | MULTICOPPER OXIDASE | 0,0857 | 2 |
| ACYPI007196-PA | RIBONUCLEASE T2 | 0,0857 | 9 |
| ACYPI009744-PA | ALDEHYDE DEHYDROGENASE | 0,0857 | 1 |
| ACYPI005526-PA | CONTACTIN, INSECT | 0,0827 | 7 |
| ACYPI009772-PA | ALKALINE PHOSPHATASE | 0,0827 | 6 |
| ACYPI000669-PA | GLUCOSE DEHYDROGENASE | 0,0797 | 2 |
| ACYPI001686-PA | EUKARYOTIC TRANSLATION INITIATION FACTOR 3 | 0,0797 | 3 |
| ACYPI005866-PA | ALDEHYDE DEHYDROGENASE | 0,0797 | 2 |
| ACYPI000575-PA | CHAPERONIN | 0,0772 | 2 |
| ACYPI005787-PA | NUCLEOLAR PROTEIN NOP56 | 0,0772 | 6 |
| ACYPI005910-PA | EGF-LIKE DOMAIN PROTEIN | 0,0772 | 5 |
| ACYPI006024-PA | ALKALINE PHOSPHATASE | 0,0772 | 4 |
| ACYPI006138-PA | UBIQUITIN-ACTIVATING ENZYME E1 | 0,0772 | 3 |
| ACYPI000622-PA | ATP SYNTHASE | 0,0747 | 2 |
| ACYPI005746-PA | VERY LONG-CHAIN ACYL-COA SYNTHETASE-RELATED | 0,0723 | 2 |
| ACYPI007757-PA | CARBOXYLESTERASE | 0,0723 | 3 |
| ACYPI009755-PA | PROTEIN DISULFIDE ISOMERASE | 0,0723 | 0 |
| ACYPI000135-PA | LEUCINE-RICH TRANSMEMBRANE PROTEIN | 0,07 | 9 |
| ACYPI000474-PA | HEAT SHOCK PROTEIN 70KDA | 0,0681 | 6 |
| ACYPI003478-PA | EXTRACELLULAR MATRIX GLYCOPROTEIN RELATED | 0,0668 | 18 |
| ACYPI009052-PA | BETA-GALACTOSIDASE | 0,0641 | 3 |
| ACYPI49694-PA | UNCHARACTERIZED | 0,0641 | 3 |
| ACYPI000145-PA | Terpenoid cyclases/Protein prenyltransferases | 0,0624 | 5 |
| ACYPI001244-PA | UNCHARACTERIZED | 0,0624 | 3 |
| ACYPI001940-PA | PHOSPHOFRUCTOKINASE | 0,0624 | 1 |
| ACYPI002583-PA | AMINOPEPTIDASE N-RELATED | 0,0624 | 7 |
| ACYPI002584-PA | ATP SYNTHASE | 0,0624 | 4 |
| ACYPI007629-PA | PAN | 0,0624 | 6 |
| ACYPI000928-PA | VON WILLEBRAND FACTOR | 0,0607 | 8 |
| ACYPI004887-PA | AMYLASE | 0,0607 | 4 |
| ACYPI004414-PA | FAMILY NOT NAMED | 0,0593 | 1 |

| | | | |
|---|---|---|---|
| ACYPI006586-PA | DIPEPTIDYL-PEPTIDASE | 0,0593 | 3 |
| ACYPI006589-PA | 3-HYDROXYACYL-COA DEHYROGENASE | 0,0593 | 2 |
| ACYPI000923-PA | GLYCOGEN DEBRANCHING ENZYME | 0,0563 | 5 |
| ACYPI008370-PA | SERINE PROTEASE-RELATED | 0,0563 | 4 |
| ACYPI000405-PA | TREHALOSE-6-PHOSPHATE SYNTHASE | 0,0551 | 4 |
| ACYPI002068-PA | LYSOSOMAL ALPHA-GLUCOSIDASE | 0,0537 | 14 |
| ACYPI008923-PA | EUKARYOTIC TRANSLATION ELONGATION FACTOR 2 | 0,0537 | 2 |
| ACYPI010100-PA | CALSYNTENIN | 0,0524 | 6 |
| ACYPI000551-PA | 26S PROTEASOME REGULATORY SUBUNIT | 0,0491 | 8 |
| ACYPI004006-PA | IMPORTIN (RAN-BINDING PROTEIN) | 0,0491 | 3 |
| ACYPI002258-PA | PROTEASE M1 ZINC METALLOPROTEASE | 0,0481 | 10 |
| ACYPI003481-PA | EUKARYOTIC TRANSLATION INITATION FACTOR 3 | 0,0452 | 5 |
| ACYPI009925-PA | EBNA2 BINDING PROTEIN P100 | 0,0452 | 5 |
| ACYPI000734-PA | GLUTAMYL AMINOPEPTIDASE | 0,0445 | 5 |
| ACYPI002950-PA | 26S PROTEASOME REGULATORY SUBUNIT | 0,0445 | 5 |
| ACYPI008056-PA | COATOMER BETA SUBUNIT | 0,0435 | 5 |
| ACYPI000303-PA | GLUTAMATE SYNTHASE | 0,0411 | 5 |
| ACYPI001659-PA | ZINC FINGER PROTEIN | 0,0411 | 12 |
| ACYPI003244-PA | Kunitz_BPTI | 0,0402 | 14 |
| ACYPI000667-PA | ALPHA-ACTININ | 0,0392 | 0 |
| ACYPI005729-PA | ACETYL-COA CARBOXYLASE | 0,0373 | 10 |
| ACYPI005219-PA | LEUCYL-TRNA SYNTHETASE | 0,0349 | 4 |
| ACYPI007409-PA | COATOMER ALPHA SUBUNIT | 0,032 | 8 |
| ACYPI002575-PA | PENTATRICOPEPTIDE REPEAT-CONTAINING PROTEIN | 0,0271 | 12 |
| ACYPI003639-PA | FnI-like domain | 0,0268 | 14 |
| ACYPI004766-PA | CARBAMOYLTRANSFERASE RELATED | 0,0198 | 7 |
| ACYPI007519-PA | HELICASE SKI2W | 0,0198 | 9 |
| ACYPI007298-PA | FATTY ACID SYNTHASE | 0,0188 | 8 |
| ACYPI001668-PA | SPECTRIN BETA CHAIN | 0,0181 | 5 |
| ACYPI004133-PA | SPECTRIN ALPHA CHAIN | 0,0148 | 5 |
| ACYPI002581-PA | DYNEIN HEAVY CHAIN | 0,0139 | 16 |

| | | | |
|---|---|---|---|
| ACYPI001617-PA | PUSHOVER/RETINOBLASTOMA-ASSOCIATED FACTOR 600 | 0,0097 | 27 |
