# Supplementary material for: Diversity in Protein Glycosylation among Insect Species
Source: PLoS One. 2011 Feb 23;6(2):e16682. doi: 10.1371/journal.pone.0016682 (PMC3044136; doi:10.1371/journal.pone.0016682)
Supplement: Table S6 — Comparative analysis of the number of annotated glycoproteins according to protein description for T. castaneum, B. mori, A. mellifera, D. melanogaster and A. pisum . (PDF) [file pone.0016682.s009.pdf]

Table S6: Comparative analysis of the number of annotated glycoproteins according to protein description for *T. castaneum, B. mori, A. mellifera, D. melanogaster* and *A. pisum* .

	Protein description	*A. pisum*	*D. melanogaster*	*A. mellifera*	*B. mori*	*T. castaneum*
1	14-3-3 protein	0	0	1	0	1
2	15-HYDROXYPROSTAGLANDIN DEHYDROGENASE	0	0	0	0	1
3	2-OXOGLUTARATE DEHYDROGENASE	0	1	1	0	1
4	3-HYDROXYACYL-COA DEHYROGENASE	1	1	1	0	1
5	4-HYDROXYPHENYLPYRUVATE DIOXYGENASE	0	0	0	0	1
6	AAA-FAMILY ATPASE	1	0	0	0	0
7	ACETYLCHOLINESTERASE	0	1	0	0	0
8	ACETYL-COA C-ACYLTRANSFERASE	1	0	1	0	1
9	ACETYL-COA CARBOXYLASE	1	0	0	0	0
10	ACID PHOSPHATASE	2	1	0	0	0
11	ACONITASE	0	0	1	0	1
12	ACTIN	2	1	2	2	1
13	ACYL-COA DEHYDROGENASE	0	0	0	0	1
14	ADENOSINE DEAMINASE	0	0	0	0	1
15	ADP-RIBOSYLATION FACTOR, ARF	0	0	1	0	0
16	ALANINE AMINOTRANSFERASE	0	0	1	0	1
17	ALCOHOL DEHYDROGENASE RELATED	0	0	1	0	0
18	ALDEHYDE DEHYDROGENASE	2	1	0	0	2
19	ALDO-KETO REDUCTASE	0	0	0	1	0
20	ALKALINE PHOSPHATASE	2	0	0	0	0
21	ALPHA,ALPHA-TREHALASE	0	0	1	1	0
22	ALPHA-ACTININ	1	0	0	0	1
23	ALPHA-AMYLASE	3	0	1	1	2
24	ALPHA-GALACTOSIDASE/ALPHA-N-ACETYLGALACTOSAMINIDASE	5	1	0	0	1
25	ALPHA-GLUCOSIDASE	2	0	0	0	0
26	ALPHA-L-FUCOSIDASE	0	1	0	0	1
27	ALPHA-MANNOSIDASE	4	2	0	0	1
28	AMINOPEPTIDASE	4	5	1	1	1
29	ANGIOPOIETIN 1 RECEPTOR	1	0	0	0	0
30	ANGIOTENSIN-CONVERTING ENZYME (DIPEPTIDYL CARBOXYPEPTIDASE)	0	2	0	1	1
31	APOLIPOPHORIN-III	0	0	0	1	1
32	ARGININE KINASE	0	1	1	0	1
33	ARYLSULFATASE B	0	0	0	0	1
34	ASPARTATE AMINOTRANSFERASE	0	1	0	0	0
35	ASPARTATE AMMONIA LYASE	0	0	1	1	1
36	ATP SYNTHASE SUBUNIT	4	2	4	1	4
37	ATP-CITRATE SYNTHASE	0	0	1	0	0
38	BETA-GALACTOSIDASE	2	1	0	0	0
39	BETA-GALACTOSIDASE RELATED	1	0	0	0	1

40	BETA-HEXOSAMINIDASE	3	1	2	0	1
41	BETA-MANNOSIDASE	0	1	0	0	0
42	BPTI_KUNITZ_2	1	1	0	0	0
43	CADHERIN	1	0	1	0	1
44	CALPONIN/TRANSGELIN	0	0	0	1	0
45	CALSYNTENIN	1	0	0	0	0
46	CARBAMOYLTRANSFERASE	1	0	0	0	0
47	CARBOXYLESTERASE	4	1	0	1	1
48	CATALASE	0	0	0	0	2
49	CATHEPSIN	1	0	1	0	1
50	CATION-TRANSPORTING ATPASE	0	0	2	0	0
51	CDP-DIACYLGLYCEROL--GLYCEROL-3-PHOSPHATE 3-PHOSPHATIDYLTRANSFERASE-RELATED	0	0	1	0	0
52	CELL ADHESION MOLECULE	3	2	0	0	0
53	CHAOPTIN	0	1	0	0	1
54	CHAPERONIN	6	0	2	0	0
55	CITRATE SYNTHASE	0	0	1	0	1
56	CLATHRIN	1	1	0	0	0
57	COATOMER ALPHA SUBUNIT	3	0	0	0	0
58	COFILIN-RELATED	1	0	0	0	0
59	COLLAGEN ALPHA CHAIN	0	0	0	0	1
60	CONTACTIN	1	1	1	0	1
61	CREATINASE	0	1	0	0	0
62	CREG1 PROTEIN	1	0	0	0	1
63	CRUMBS-RELATED	0	1	0	0	0
64	Crustacean CHH/MIH/GIH neurohormone	0	1	0	0	0
65	C-TYPE LECTIN DOMAIN CONTAINING PROTEIN	1	4	0	0	0
66	CYSTEINE PROTEASE FAMILY C1-RELATED	1	1	0	0	0
67	CYSTEINE-RICH SECRETORY PROTEIN (CRISP/SCP/TPX1)-RELATED	0	1	0	0	0
68	CYTOCHROME C OXIDASE	0	0	0	0	1
69	CYTOCHROME P450 SUBFAMILY 4G	0	0	1	0	0
70	DEAD-BOX PROTEIN	1	0	0	0	0
71	DEHYDRODOLICHYL DIPHOSPHATE SYNTHASE	0	0	0	1	0
72	DENDRITIC CELL PROTEIN GA17	1	0	0	0	0
73	DEOXYRIBONUCLEASE II	1	0	0	0	0
74	DIHYDROLIPOAMIDE ACETYL/SUCCINYL-TRANSFERASE-RELATED	0	0	0	0	1
75	DIHYDROPYRIDINE-SENSITIVE L-TYPE CALCIUM CHANNEL	0	1	1	0	0
76	DIPEPTIDYL-PEPTIDASE	1	0	0	0	0
77	DISULFIDE OXIDOREDUCTASE	0	0	0	1	0
78	DOMON	0	1	0	0	0
79	DUF3421	0	2	0	1	0
80	DYNEIN HEAVY CHAIN	1	0	0	0	0
81	EBNA2 BINDING PROTEIN	1	0	0	0	0
82	EGF-LIKE DOMAIN PROTEIN	3	1	0	0	0

83	ELECTRON TRANSPORT OXIDOREDUCTASE	0	0	2	0	1
84	ELONGATION FACTOR 1-ALPHA (EF-1-ALPHA)	1	0	1	0	1
85	ENOLASE	0	0	0	1	0

86	ENOLASE-PHOSPHATASE E-1	0	0	1	0	0
87	ENOYL-COA HYDRATASE-RELATED	0	0	0	0	1
88	EUKARYOTIC TRANSLATION INITATION FACTOR	8	0	1	0	0
89	EXTRACELLULAR MATRIX GLYCOPROTEIN RELATED	2	0	0	0	0
90	FAD NADPH DEHYDROGENASE/OXIDOREDUCTASE	1	0	0	0	0
91	FASCICLIN I (FAS I)	0	0	0	0	1
92	FASCICLIN II (FAS II)	0	0	1	0	1
93	FATTY ACID SYNTHASE	3	0	0	0	0
94	FERRITIN SUBUNIT 1	0	0	0	1	1
95	FIBRINOGEN C	0	1	0	0	0
96	FIBRINOGEN-RELATED	0	1	0	0	0
97	FIBRONECTIN TYPE III	0	5	0	0	0
98	FNI-LIKE	1	0	0	0	0
99	FRUCTOSE-1,6-BISPHOSPHATASE-RELATED	0	0	0	0	1
100	FRUCTOSE-BISPHOSPHATE ALDOLASE	1	0	0	0	1
101	FUMARYLACETOACETATE HYDROLASE	0	0	0	0	1
102	GALACTOSE-SPECIFIC C-TYPE LECTIN	2	2	0	0	0
103	GLUCOSE DEHYDROGENASE	2	1	0	0	0
104	GLUCOSE-6-PHOSPHATE ISOMERASE	0	0	1	0	0
105	GLUTAMATE CARBOXYPEPTIDASE	0	0	0	1	0
106	GLUTAMATE DEHYDROGENASE	0	0	1	0	1
107	GLUTAMATE SEMIALDEHYDE DEHYDROGENASE	0	0	1	0	0
108	GLUTAMINE SYNTHETASE	3	0	0	0	0
109	GLUTAMYL AMINOPEPTIDASE	1	0	0	0	0
110	GLUTATHIONE PEROXIDASE	0	0	1	0	0
111	GLUTATHIONE S-TRANSFERASE	0	0	0	0	1
112	GLYCERALDEHYDE 3-PHOSPHATE DEHYDROGENASE	1	0	1	0	1
113	GLYCEROL-3-PHOSPHATE DEHYDROGENASE	0	0	2	0	0
114	GLYCOGEN DEBRANCHING ENZYME	2	1	1	0	0
115	GLYCOGEN PHOSPHORYLASE	1	0	1	1	1
116	GLYCOGEN SYNTHASE	0	1	0	0	0
117	GLYCOSIDE HYDROLASES	0	0	0	2	0
118	GLYPICAN	1	1	0	0	0
119	GRAM-NEGATIVE BACTERIA BINDING PROTEIN 1	0	0	0	1	0
120	GTP-BINDING PROTEIN SAR1	0	0	1	0	0
121	HEAT SHOCK PROTEIN	5	0	4	1	7
122	HELICASE	1	0	0	0	0
123	HEPARAN N-SULFATASE	0	0	1	0	1
124	HISTONE	1	0	0	0	1
125	HOMOGENTISATE 1,2-DIOXYGENASE	0	0	0	0	1
126	HOST CELL FACTOR-RELATED	0	0	0	1	0
127	IMMUNOGLOBULIN DOMAIN SUPERFAMILY (SENSORY GUIDANCE PROTEIN)	0	0	0	0	2

128	IMPORTIN (RAN-BINDING PROTEIN)	1	0	0	0	0
129	INORGANIC PYROPHOSPHATASE	0	0	0	0	1
130	INOSINE-5-MONOPHOSPHATE DEHYDROGENASE	1	0	0	0	0
131	INS ALLERGEN RP	0	1	0	0	0
132	INSECT HEMOCYANIN-RELATED	0	1	2	2	6
133	INTER-ALPHA-TRYPSIN INHIBITOR HEAVY CHAIN	0	0	0	0	1
134	ISOCITRATE DEHYDROGENASE	1	2	1	0	0
135	JUVENILE HORMONE BINDING PROTEIN	1	0	0	1	0
136	JUVENILE HORMONE ESTERASE	0	0	0	0	1
137	KETOACID-COENZYME A TRANSFERASE	0	0	1	0	0
138	LAMININ	5	4	0	1	4
139	L-ASPARAGINASE	1	0	0	0	0
140	LEUCINE AMINOPEPTIDASE	0	0	0	0	1
141	LEUCINE-RICH TRANSMEMBRANE PROTEIN	15	1	0	0	4
142	LEUCYL-TRNA SYNTHETASE	1	0	0	0	0
143	LIPASE	0	1	0	1	0
144	LIPOPROTEIN	0	2	0	3	0
145	LONG-CHAIN-FATTY-ACID COA LIGASE	0	0	0	1	0
146	LOW DENSITY LIPOPROTEIN RECEPTOR	1	1	1	0	2
147	L-XYLULOSE REDUCTASE/LUNG CARBONYL REDUCTASE [NADPH]	1	0	0	0	0
148	LYSOSOMAL ACID LIPASE-RELATED	0	0	0	0	1
149	MACROGLOBULIN/COMPLEMENT	0	2	1	0	0
150	MACROPHAGE MIGRATION INHIBITORY FACTOR RELATED	1	0	0	0	0
151	MALATE AND LACTATE DEHYDROGENASE	0	0	1	0	0
152	MALIC ENZYME	0	0	0	0	2
153	METALLOPROTEASE	1	4	0	0	0
154	MIDLINE FASCICLIN	0	1	0	0	0
155	MITOCHONDRIAL CARRIER PROTEIN	1	0	1	0	0
156	MITOCHONDRIAL PROCESSING PEPTIDASE BETA SUBUNIT	0	0	1	0	1
157	MUCIN	0	1	2	2	0
158	MULTICOPPER OXIDASE	1	0	0	0	1
159	MULTIPLE INOSITOL POLYPHOSPHATE PHOSPHATASE	0	0	0	0	1
160	MYOSIN	1	1	0	2	0
161	NADH-UBIQUINONE OXIDOREDUCTASE	0	0	2	0	0
162	NEPRILYSIN	1	0	1	0	0
163	NEUREXIN IV	0	1	0	0	0
164	NEUROTRACTING/LSAMP/NEUROTRIMIN/OBCAM RELATED CELL ADHESION MOLECULE	0	0	0	0	1
165	NICOTINATE PHOSPHORIBOSYLTRANSFERASE	0	1	0	0	0
166	NITRIC OXIDE SYNTHASE	0	0	1	0	0
167	NUCLEAR PROTEIN SKIP-RELATED	0	0	1	0	0
168	NUCLEOLAR PROTEIN NOP56	1	0	0	0	0
169	ORNITHINE AMINOTRANSFERASE	1	0	0	0	0

170	OXIDASE/PEROXIDASE	1	0	0	0	0
171	PAPILIN	1	0	0	0	0
172	PENTATRICOPEPTIDE REPEAT-CONTAINING PROTEIN	1	0	0	0	0
173	PERIOSTIN-RELATED	0	1	0	0	0
174	PERIPLASMIC BINDING PROTEIN-LIKE II	0	0	0	0	1
175	PEROXIREDOXIN	0	0	1	1	1
176	PHOSPHATIDYLCHOLINE-STEROL ACYLTRANSFERASE-RELATED	0	0	1	0	0
177	PHOSPHATIDYLINOSITOL TRANSFER PROTEIN/RETINAL DEGENERATION B PROTEIN	0	0	1	0	0
178	PHOSPHOFRUCTOKINASE	1	1	1	0	0
179	PHOSPHOGLYCERATE KINASE	0	0	1	0	0
180	PLASMA MEMBRANE CA-ATPASE B2	0	0	1	0	0
181	PLC-like phosphodiesterases	0	1	0	0	0
182	PROCOLLAGEN/CELL ADHESION MOLECULE RELATED	2	0	0	0	1
183	PROHIBITIN	0	0	1	0	0
184	PROLYL 4-HYDROXYLASE ALPHA SUBUNIT	1	0	0	0	0
185	PROLYLCARBOXYPEPTIDASE/PRCP	1	1	0	0	0
186	PROTEASE FAMILY C26 GAMMA-GLUTAMYL HYDROLASE	2	0	0	0	0
187	PROTEASE FAMILY M28 PLASMA GLUTAMATE CARBOXYPEPTIDASE-RELATED	1	0	0	0	1
188	PROTEASE M1 ZINC METALLOPROTEASE	3	1	0	0	0
189	PROTEASE REGULATORY SUBUNIT	10	1	0	0	0
190	PROTEASE S28 PRO-X CARBOXYPEPTIDASE-RELATED	2	1	0	0	1
191	PROTEASE T2 ASPARAGINASE	2	0	0	0	0
192	PROTEASOME SUBUNIT	2	0	0	0	2
193	PROTEIN DISULFIDE ISOMERASE	3	1	0	1	1
194	Protein kinase-like (PK-like)	0	1	0	0	0
195	PROTEIN TYROSINE PHOSPHATASE 69D, DROME	0	1	0	0	0
196	PTD012 PROTEIN	0	0	0	1	0
197	PURINE BIOSYNTHESIS PROTEIN 6, PUR6	0	0	0	0	1
198	PUROMYCIN-SENSITIVE AMINOPEPTIDASE	0	0	1	0	0
199	PUR-TRANSCRIPTIONAL ACTIVATOR	0	0	0	0	1
200	PUSHOVER/RETINOBLASTOMA-ASSOCIATED FACTOR 600	1	0	0	0	0
201	PYRUVATE CARBOXYLASE	0	0	1	0	0
202	PYRUVATE DEHYDROGENASE	0	0	2	0	0
203	PYRUVATE DEHYDROGENASE E1 COMPONENT, BETA SUBUNIT	0	0	0	0	1
204	QUIESCIN Q6-RELATED SULFHYDRYL OXIDASE	0	0	0	0	1
205	RECEPTOR FOR ACTIVATED PROTEIN KINASE C (RACK1)	0	1	1	0	0
206	RAS-RELATED GTPASE	1	0	0	0	0
207	RETICULON-RELATED	0	1	0	0	0
208	RETINOID-INDUCIBLE SERINE CARBOXYPEPTIDASE (SERINE CARBOXYPEPTIDASE 1)	0	0	1	0	0
209	REVERSE TRANSCRIPTASE	0	0	0	0	1
210	RIBONUCLEASE T2	1	0	0	0	0
211	RIBOPHORIN I	0	0	1	0	0
212	RIBOSOMAL PROTEINS	18	14	10	5	22

213	RIBOSOME BIOGENESIS PROTEIN BRIX	0	0	0	1	0
214	RNA-BINDING PROTEIN	0	1	0	0	0
215	SARCALUMENIN	0	0	1	0	0
216	SECRETED GLUCOSIDASE-RELATED	1	0	0	0	0
217	SECRETED SUGAR HYDROLASE	0	0	1	0	0
218	SEMAPHORIN	0	1	0	0	0
219	SERINE CARBOXYPEPTIDASE	2	1	0	0	1
220	SERINE PROTEASE INHIBITOR, SERPIN	4	1	0	1	4
221	SERINE PROTEASE-RELATED	2	7	0	7	0
222	SERINE/THREONINE PROTEIN PHOSPHATASE 2A REGULATORY SUBUNIT A	1	1	0	0	0
223	SHORT-CHAIN DEHYDROGENASE-RELATED	0	0	0	0	1
224	SOLUTE CARRIER FAMILY 27 (FATTY ACID TRANSPORTER)	0	0	1	0	0
225	SPECTRIN ALPHA CHAIN	1	0	0	0	0
226	SPECTRIN BETA CHAIN	1	0	1	0	0
227	SPECTRIN-LIKE CELL STRUCTURE PROTEIN	0	0	1	0	0
228	SPHINGOMYELIN PHOSPHODIESTERASE 1, 2	0	0	0	0	1
229	SUCCINATE DEHYDROGENASE 2 FLAVOPROTEIN SUBUNIT	0	1	0	0	0
230	SUCCINATE DEHYDROGENASE IRON-SULFUR PROTEIN	0	0	1	0	0
231	SUCCINATE SEMIALDEHYDE DEHYDROGENASE	0	0	0	0	1
232	SUCCINYL-COA SYNTHETASE BETA CHAIN	0	1	1	0	0
233	SUCROSE-6-PHOSPHATE HYDROLASE	0	0	0	1	0
234	SUSHI DOMAIN CONTAINING PROTEIN	0	0	0	0	1
235	TBC1 DOMAIN FAMILY MEMBER GTPASE-ACTIVATING PROTEIN	0	0	0	0	1
236	TCTPROTEIN	1	0	0	0	0
237	TERPENOID CYCLASES	1	1	0	0	0
238	THIOREDOXIN_2	0	1	0	0	0
239	TRANSFERRIN	0	0	0	1	0
240	TRANSKETOLASE	1	0	1	0	1
241	TRANSMEMBRANE PROTEASE, SERINE 2	0	0	1	0	0
242	TREHALOSE	1	0	0	0	0
243	TREHALOSE-6-PHOSPHATE SYNTHASE	1	1	1	0	0
244	TROPOMYOSIN	1	0	0	1	1
245	TUBULIN	4	4	4	1	0
246	UBIQUINOL-CYTOCHROME C REDUCTASE COMPLEX CORE PROTEIN 2	0	0	0	0	1
247	UBIQUITIN-ACTIVATING ENZYME E1	1	0	0	0	0
248	UBIQUITIN-CONJUGATING ENZYME E2	0	0	1	0	0
249	UDP-GLUCOSE GLYCOPROTEIN:GLUCOSYLTRANSFERASE	0	0	0	1	1
250	UDP-N-ACETYLGLUCOSAMINE--PEPTIDE N-ACETYLGLUCOSAMINYLTRANSFERASE 110 KDA SUBUNIT	1	0	0	0	0
251	UDP-N-ACTEYLGLUCOSAMINE PYROPHOSPHORYLASE 1	0	0	0	0	1
252	UNCHARACTERIZED	15	10	3	2	10
253	VERY LONG-CHAIN ACYL-COA SYNTHETASE-RELATED	1	0	0	0	0
254	VITELLOGENIN-RELATED	1	2	0	0	4

255	VOLTAGE-DEPENDENT ANION-SELECTIVE CHANNEL	0	0	1	0	0
256	VON WILLEBRAND FACTOR, TYPE A DOMAIN CONTAINING	2	0	0	0	0
257	V-TYPE ATP SYNTHASE ALPHA CHAIN	0	0	2	1	1
258	V-TYPE ATP SYNTHASE BETA CHAIN	0	0	0	1	0
259	YELLOW PROTEIN-RELATED	1	0	0	0	0
260	ZINC FINGER PROTEIN	1	0	0	0	1
261	ZN/RING FINGER PROTEIN 2	0	1	0	0	0
