# Supplementary material for: Diversity in Protein Glycosylation among Insect Species
Source: PLoS One. 2011 Feb 23;6(2):e16682. doi: 10.1371/journal.pone.0016682 (PMC3044136; doi:10.1371/journal.pone.0016682)
Supplement: Table S7 — WU-BLAST analysis to search for proteins homologous to O -mannosyltransferases from Drosophila melanogaster POMT1 (Genbank accession No NP_524025.2) and POMT2 (Genbank accession No NP_569858.1). (PDF) [file pone.0016682.s010.pdf]

Table S7: WU-BLAST analysis to search for proteins homologous to O-mannosyltransferases from Drosophila melanogaster POMT1 (Genbank accession No NP_524025.2) and POMT2 (Genbank accession No NP_569858.1).

**Accession number**	**Insect species**	**Annotation**	**% Identity**	**% Similarity**
Homologous to POMT1				
NP_524025.2	*Drosophila melanogaster*	protein O-mannosyltransferase 1 (POMT1)	100	100
BGIBMGA003152	*Bombyx mori*	predicted: derived from silkbase cDNA database	69	83
XP_623815.2	*Apis mellifera*	predicted: similar to rotated abdomen	55	71
XP_971065.1	*Tribolium castaneum*	predicted: similar to rotated abdomen	54	71
XP_001949721.1	*Acyrthosiphon pisum*	predicted: similar to AGAP010784-PA	52	70
Homologous to POMT2				
NP_569858.1	*Drosophila melanogaster*	protein O-mannosyltransferase 2 (POMT2)	100	100
BGIBMGA005070	*Bombyx mori*	predicted: derived from silkbase cDNA database	66	78
XP_973439.1	*Tribolium castaneum*	predicted: similar to protein mannosyltransferase	63	75
XP_623973.1	*Apis mellifera*	predicted: similar to protein O-mannosyltransferase 2	62	75
XP_001946715.1	*Acyrthosiphon pisum*	predicted: similar to mannosyltransferase	55	70
